# Applications and Challenges of GRACE and GRACE Follow-On Satellite Gravimetry

**DOI:** 10.1007/s10712-021-09685-x

**Published:** 2022-01-09

**Authors:** Jianli Chen, Anny Cazenave, Christoph Dahle, William Llovel, Isabelle Panet, Julia Pfeffer, Lorena Moreira

**Affiliations:** 1 Center for Space Research, University of Texas at Austin, Austin, TX 78759 USA; 2 Legos/CNES, 14 Avenue Edouard Belin, 31400 Toulouse, France; 3 GFZ German Research Centre for Geosciences, 14473 Potsdam, Germany; 4 LOPS, University of Brest/IFREMER/IRD/CNRS, 29280 Brest, France; 5 Université de Paris, IPGP/CNRS/IGN, 75005 Paris, France; 6 Magellium, 31520 Ramonville Saint-Agne, France; 7 International Space Science Institute, Hallerstrasse 6, 3012 Bern, Switzerland

**Keywords:** GRACE, GRACE-FO, Satellite gravimetry, Gravity, Mass change, Application, Challenge

## Abstract

Time-variable gravity measurements from the Gravity Recovery and Climate Experiment (GRACE) and GRACE Follow-On (GRACE-FO) missions have opened up a new avenue of opportunities for studying large-scale mass redistribution and transport in the Earth system. Over the past 19 years, GRACE/GRACE-FO time-variable gravity measurements have been widely used to study mass variations in different components of the Earth system, including the hydrosphere, ocean, cryosphere, and solid Earth, and significantly improved our understanding of long-term variability of the climate system. We carry out a comprehensive review of GRACE/GRACE-FO satellite gravimetry, time-variable gravity fields, data processing methods, and major applications in several different fields, including terrestrial water storage change, global ocean mass variation, ice sheets and glaciers mass balance, and deformation of the solid Earth. We discuss in detail several major challenges we need to face when using GRACE/GRACE-FO time-variable gravity measurements to study mass changes, and how we should address them. We also discuss the potential of satellite gravimetry in detecting gravitational changes that are believed to originate from the deep Earth. The extended record of GRACE/GRACE-FO gravity series, with expected continuous improvements in the coming years, will lead to a broader range of applications and improve our understanding of both climate change and the Earth system.

## **Article Highlights**

- A comprehensive review of major applications of GRACE/GRACE-FO satellite gravimetry
- Discussions in detail of some major challenges in GRACE/GRACE-FO mass change estimation
- Discussions of the potential detection of deep Earth signals by GRACE/GRACE-FO gravimetry

## Introduction

Since the Gravity Recovery and Climate Experiment (GRACE) was launched in March 2002, satellite gravimetry has brought a new era of studying global mass variation and redistribution through measuring the time-variable gravity field with unprecedented accuracy (Tapley et al. 2019). GRACE is a twin satellites mission utilizing a state-of-the-art technique to map variations of the global gravity field by accurately tracking variations of inter-satellite range between the two satellites via a *K*-band ranging (KBR) system. GRACE time-variable gravity measurements have been widely used in studies of mass variation in different components of the climate system, including regional to global scale terrestrial water storage (TWS) change, flood and drought detection, groundwater depletion, water storage change in snow and surface reservoirs, polar ice sheets and mountain glacier ice-mass change, global sea level change, and others (Tapley et al. 2019). GRACE satellite gravimetry provides a unique tool for studying solid Earth deformation due to large earthquakes (Han et al. 2006; Li et al. 2016) and Glacial Isostatic Adjustment (GIA) (Tamisiea et al. 2007; Riva et al. 2009). GRACE-derived TWS change can also be used to investigate terrestrial water effects on the carbon cycle variability at global to regional scales (Humphrey et al. 2018).

After well exceeding the planned five-year life span, GRACE was decommissioned in November 2017, and the GRACE Follow-On (GRACE-FO) mission was launched in May 2018 to continue the endeavor. GRACE-FO is basically a duplicate of GRACE with a similar satellite orbit configuration and improved system design. In addition, the GRACE-FO satellites also carry a Laser Ranging Interferometer (LRI) for experimenting purposes (LRI is designed for future generations of satellite gravimetry missions). The combined GRACE and GRACE-FO observations provide an extended record of precise measurements of the Earth’s time-variable gravity field, which will continuously improve our understanding of mass variations in the climate system, especially at long-term time scales. So far, over three years of GRACE-FO time-variable gravity solutions with comparable accuracy as from GRACE have been released (Landerer et al. 2020). There is an about 1-year gap (July 2017–May 2018) between the GRACE and GRACE-FO missions, which is not ideal, but would not affect most of related applications focusing on seasonal and long-term time scales. Initial analyses using GRACE-FO data show encouraging results with accuracy mostly consistent with pre-launch expectations (Landerer et al. 2020; Velicogna et al. 2020; Boergens et al. 2020).

Despite the tremendous success of GRACE and GRACE-FO (noted as GRACE/GRACE-FO hereafter unless for separate discussions), accurate quantification of mass variations using GRACE/GRACE-FO gravity measurements has been challenging due to its coarse spatial resolution and limited accuracy. In addition to the KBR system, each GRACE/GRACE-FO satellite is equipped with a SuperSTAR Accelerometer (ACC), GPS receiver/antenna, Star Cameras, and Laser Retro Reflectors to complement the science instruments. The GRACE/GRACE-FO Science Data System (SDS) consisting of the Center for Space Research at the University of Texas at Austin (CSR), NASA’s Jet Propulsion Laboratory (JPL), and the German Research Centre for Geosciences (GFZ) uses the ranging and ancillary data to estimate a new gravity field every month, in the form of corrections to a background gravity model used in the data processing procedure (Bettadpur 2018; Yuan 2018; Dahle et al. 2019). Apart from the SDS, several other processing centers also generate monthly gravity field solutions, many of which contribute to a recently established Combination Service for Time-variable Gravity Fields (COST-G; Jäggi et al. 2020). The spatial resolution and accuracy of GRACE/GRACE-FO time-variable gravity solutions depend on many factors, including (but not limited to) the accuracy of KBR and ACC measurements, uncertainty of geophysical background models (ocean tides, solid Earth tides, atmospheric tides, atmosphere and ocean models), orbits of the satellites (altitude, inclination and inter-satellite distance), data editing and calibration (satellite measurements to gravity field) procedures. The orbit configurations of GRACE/GRACE-FO satellites, with initial altitudes of ~ 500 km and inter-satellite distance of ~ 220 km, place some fundamental limitations on the spatial resolution of GRACE-derived gravity (or mass) changes on Earth’s surface, so that resolution is not expected to be better than a few hundred km.

In addition, GRACE/GRACE-FO high-degree and -order spherical harmonic (SH) coefficients are dominated by noise, and spatial filtering and/or smoothing are needed in order to suppress the noise and extract meaningful mass change signals. The applied spatial filtering (i.e., down-weighting of high-degree and -order coefficients) further degrades GRACE/GRACE-FO spatial resolution and creates an additional spatial leakage error and bias in GRACE/GRACE-FO estimates. The leakage error reflects the attenuation of the amplitude of the signal and the spread of the signal into neighboring areas as the consequence of spatial smoothing. Even though improved geophysical background models and data processing methods can somewhat reduce these limitations, errors remain, especially at basin and regional scales. GRACE/GRACE-FO very low degree SH coefficients, especially the degree-2 zonal coefficient $\Delta C_{2,0}$ (and also $\Delta C_{3,0}$ during late stage of GRACE and the GRACE-FO period) are also poorly estimated. The late stage GRACE and GRACE-FO are both operated with only one ACC functioning properly, which introduces significantly large noise in the $\Delta C_{2,0}$ and $\Delta C_{3,0}$ coefficients (Landerer et al. 2020). Correctly defining the reference frame also affects GRACE/GRACE-FO estimated mass change at the global scale, because the Earth gravity field and mass change are defined in different reference frames. As GRACE/GRACE-FO can only measure the total mass change of a given area, to quantify mass change associated with the climate system, solid Earth contributions (e.g., the GIA effect) need to be removed from GRACE/GRACE-FO gravity solutions using model predictions. This brings in another source of uncertainty.

The main objectives of this study are to (1) provide a basic introduction of gravity field theory and mass inversion from GRACE/GRACE-FO time-variable gravity solutions, (2) discuss major challenges in GRACE/GRACE-FO mass change estimation and how we could address them, and (3) demonstrate GRACE/GRACE-FO potential applications in various components of the climate system and solid Earth deformation. We will also discuss the possibility of satellite gravimetry to detect deep Earth gravitational change signal. In theory, satellite gravimetry is unable to separate contributions to observed gravity change from surface and deep Earth sources, due to the non-uniqueness of mass inversion (Chao 2005). However, when combined with information from other independent sources or knowledge, it is possible to detect certain signals likely originated from the deep Earth using GRACE/GRACE-FO satellite gravimetry measurements.

## Time-Variable Gravity Field and Mass Change

### Gravitational Field

Governed by Newton’s universal law of gravitation, the gravitational potential (geopotential) V at a given point in space (***r***) produced by a body of internal mass distribution is given by (Chao 2005),

1 $$V\left( {\mathbf{r}} \right) = G\iiint_{{A_{0} }} {\frac{{\rho \left( {{\mathbf{r}}_{0} } \right)}}{{\left| {{\mathbf{r}} - {\mathbf{r}}_{0} } \right|}}{\text{d}}A}$$

in which $G$ is the universal gravitational constant, and ${\varvec{r}}$ is the position vector represented by radius *r*, co-latitude θ, and longitude λ point in spherical coordinates. $\rho \left( {{\varvec{r}}_{0} } \right)$ and ${\text{d}}A$ are mass density and volume element at position ${\varvec{r}}_{0} ,$ and the integration is over the entire internal mass body ($A_{0}$).

For the Earth gravity field, the above equation can be conveniently expressed as spherical harmonic expansion as (Kaula 1966),

2 $$U\left( {r,\theta ,\lambda } \right) = \frac{GM}{a}\mathop \sum \limits_{l = 0}^{\infty } \mathop \sum \limits_{m = 0}^{l} \left( \frac{a}{r} \right)^{l + 1} P_{lm} \left( {cos\theta } \right)(C_{lm} \cos m\lambda + S_{lm} \sin m\lambda )$$

in which, $M$ is the mass, *a* the mean equatorial radius of the Earth, and $P_{lm}$ the 4π-normalized Legendre function. $C_{lm}$ and $S_{lm}$ are SH coefficients of degree *l* and order *m.* $C_{lm}$ and $S_{lm}$ are related to the internal density distribution as,

3 $$\left( {\begin{array}{*{20}l} {C_{lm} } \hfill \\ {S_{lm} } \hfill \\ \end{array} } \right) = \frac{1}{{\left( {2l + 1} \right)Ma^{l} }}\iiint_{{V_{0} }} {\rho \left( {\mathbf{r}} \right)r^{l} P_{lm} \left( {\cos \theta } \right)}\left( {\begin{array}{*{20}l} {\cos m\lambda } \hfill \\ {\sin m\lambda } \hfill \\ \end{array} } \right)dV$$

The above equations describe the Earth’s static gravity field from the mass distribution in the Earth system. Given a 3D internal mass redistribution $\rho \left( {\varvec{r}} \right)$, the gravity (or geopotential) SH coefficients at any point in the space can be uniquely determined by 3D integration over the internal mass body. However, the inversion of mass density change $\rho \left( {\varvec{r}} \right)$ from observed time-variable gravity change $C_{lm}$ and $S_{lm}$ is non-unique. The non-uniqueness of mass inversion from gravity field was discussed in detail in Chao (2005). Temporal variations of mass distribution in the Earth system will also affect the gravity field, causing time-variable gravity changes that can be observed by GRACE/GRACE-FO. In theory, to describe the full spectrum of the gravity field, one would need SH coefficients $C_{lm}$ and $S_{lm}$ up to degree and order of infinity. Limited by the number of observations or spatial resolutions of observational techniques, observed gravity fields, either static or time-variable, are always expressed by SH coefficients up to a certain degree and order, which typically define the spatial resolution of the gravity fields when no noise or error exists. More discussions of gravity field resolution can be found in Devaraju and Sneeuw (2015) in a context of satellite gravimetry.

### GRACE/GRACE-FO Time-Variable Gravity Field and Mass Variation

GRACE/GRACE-FO time-variable gravity solutions are provided by the three GRACE/GRACE-FO SDS data processing centers CSR, JPL, and GFZ, and other institutions, e.g., TU Graz in Austria, Tongji University in China, the International Combination Service for Time-variable Gravity Fields (COST-G), etc. The current SDS Release 6 (RL06) solutions are expressed in the form of SH coefficients up to a maximum degree and order of 60 (degree and order 96 products are also available). These monthly solutions are routinely distributed by NASA’s Physical Oceanography Distributed Active Archive Center (PODAAC, http://podaac.jpl.nasa.gov/grace/) and GFZ’s Information System and Data Center (ISDC, http://isdc.gfz-potsdam.de/grace-isdc/) with a typical latency of less than 60 days. The truncation of GRACE/GRACE-FO gravity solutions at degree and order 60 (or 96) is mainly determined by GRACE/GRACE-FO satellite orbit configuration (satellite altitude and inter-satellite distance).

Atmospheric and dynamical oceanic mass variations are largely removed during GRACE/GRACE-FO gravity field processing by using a non-tidal atmosphere and ocean de-aliasing model (Dobslaw et al. 2017a). The high-frequency atmospheric and oceanic signals, if not removed, will introduce artifacts (at lower frequencies) in the GRACE/GRACE-FO monthly fields. For each monthly GRACE/GRACE-FO gravity solution (the so-called GSM product), the processing centers provide some supplementary datasets that contain the monthly means of the removed atmosphere and ocean de-aliasing model. For example, the supplementary GAC product represents the combined non-tidal atmospheric and oceanic mass changes removed from GRACE/GRACE-FO GSM solutions. Please see Dobslaw et al. (2017b) for detailed definitions of the supplementary fields (e.g., GAC, GAD, and GAA). These removed signals need to be restored to GRACE/GRACE-FO GSM products for certain applications (e.g., total gravity change over land or bottom pressure change over the ocean).

Gravity change as observed by GRACE/GRACE-FO represents integrated contributions from 3-dimensional (3D) mass redistribution in the Earth system, from the top of the atmosphere to the deep solid Earth, which can be described by a variable form of Eq. (3) as,

4 $$\left( {\begin{array}{*{20}l} {\Delta C_{lm} \left( t \right)} \hfill \\ {\Delta S_{lm} \left( t \right)} \hfill \\ \end{array} } \right) = \frac{1}{{\left( {2l + 1} \right)Ma^{l} }}\iiint_{{V_{0} }} \Delta \rho \left( {{\varvec{r}},t} \right)r^{l} P_{lm} \left( {cos\theta } \right)\left( {\begin{array}{*{20}l} {\cos m\lambda } \hfill \\ {\sin m\lambda } \hfill \\ \end{array} } \right){\text{d}}V$$

Similar to the static gravity field, time-variable gravity changes $\Delta C_{lm} \left( t \right)$ and $\Delta S_{lm} \left( t \right)$ can be uniquely determined by 3D integration of mass density change $\Delta \rho \left( {{\varvec{r}},t} \right)$ over the internal mass body (i.e., from the center of the Earth up to the satellite altitude for satellite gravimetry), but the inversion of mass density change $\Delta \rho \left( {{\varvec{r}},t} \right)$ from observed time-variable gravity change $\Delta C_{lm} \left( t \right)$ and $\Delta S_{lm} \left( t \right)$ is non-unique (Chao 2005).

If we can assume that, at decadal or shorter time scales, mass variations in the Earth system mainly occur as air and water mass redistributions in the atmosphere, ocean, hydrosphere, and cryosphere (i.e., the geophysical fluids envelope), surface mass density change $\Delta \sigma \left( {\theta ,\lambda } \right)$ can be readily estimated from GRACE/GRACE-FO observed time-variable gravity solutions as (Chao et al. 1987; Wahr et al. 1998),

5 $$\Delta \sigma \left( {\theta ,\lambda } \right) = \frac{M}{{4\pi a^{2} }}\mathop \sum \limits_{l = 0}^{\infty } \mathop \sum \limits_{m = 0}^{l} \frac{2l + 1}{{1 + k_{l} }}P_{lm} \left( {cos\theta } \right)\left( {\Delta C_{lm} \cos m\lambda + \Delta S_{lm} \sin m\lambda } \right)$$

where $k_{l}$ are the load Love numbers accounting for load deformation of the solid Earth due to changes of surface loads. The above equation simplifies the mass inversion on to a 2-dimensional (2D) spherical shell on the Earth surface.

The degree-0 SH coefficients ($\Delta C_{0,0}$) reflect variations of the total mass of the Earth system, and the degree-1 coefficients ($\Delta C_{1,1}$,$\Delta S_{1,1}$, and $\Delta C_{1,0}$) represent the three components of geocenter motion ($\Delta X_{{{\text{gc}}}}$, $\Delta Y_{{{\text{gc}}}}$, and $\Delta Z_{{{\text{gc}}}}$). Considering that the total mass of the Earth system is a constant and the gravity field is commonly defined in the center of mass (CM) reference frame, GRACE/GRACE-FO gravity SH coefficients are provided for degree 2 and above.

### Major Challenges in GRACE/GRACE-FO Mass Estimation

The high-degree and -order SH coefficients observed by GRACE/GRACE-FO are dominated by noise, characterized by strong longitudinal stripes and other errors. Swenson and Wahr (2006) indicated that the longitudinal stripes in GRACE gravity solutions appeared to be related to the correlation between the even and odd degree pairs of GRACE SH coefficients of the same order, and can be mostly removed using a decorrelation filtering. Other noise in the high-degree and -order coefficients can be further suppressed by Gaussian smoothing, i.e., down-weighting contributions from high-degree coefficients by applying the Gaussian spectral weight as a function of degree *l* ($W_{l}$) to $\Delta C_{lm}$ and $\Delta S_{lm}$ in Eq. (5) as,

6 $$\Delta \sigma \left( {\theta ,\lambda } \right) = \frac{M}{{4\pi a^{2} }}\mathop \sum \limits_{l = 0}^{\infty } \mathop \sum \limits_{m = 0}^{l} \frac{2l + 1}{{1 + k_{l} }}W_{l} P_{lm} \left( {{\text{cos}}\theta } \right)\left( {\Delta C_{lm} \cos m\lambda + \Delta S_{lm} \sin m\lambda } \right)$$

Equations for calculating the Gaussian weights as a function of degree *l* for a given spatial radius (e.g., 300 km) are provided in Wahr et al. (1998). The 2-step spatial filtering, decorrelation plus Gaussian smoothing at certain spatial radius has been widely used in GRACE/GRACE-FO related applications. Some other filtering methods have also been developed over the years to help reduce the stripes and other noise, e.g., the empirical orthogonal function filter (Wouters and Schrama 2007), non-symmetric filter based on GRACE variance–covariance matrix (Klees et al. 2008), and DDK filter mimicking a regularization of the GRACE normal equations (Kusche et al. 2009). In the following, we summarize some major challenges in GRACE/GRACE-FO mass change estimation.

#### Leakage Bias

One of the biggest challenges in GRACE/GRACE-FO applications is the coarse spatial resolution of GRACE/GRACE-FO derived mass change fields (at best about three hundred km). GRACE/GRACE-FO spatial resolution is mainly controlled by two factors: (1) the availability of limited degree and order (60 or 96 in this case) of SH coefficients, and (2) the attenuation effect due to spatial filtering and smoothing applied to the GRACE/GRACE-FO fields. Limitation to degrees below 60 or 96 and filtering are necessary due to the low sensitivity of the GRACE/GRACE-FO measurements to high-degree components (Eq. 4). The truncation of SH coefficients (e.g., at degrees and orders of 60 or 96) and spatial filtering lead to attenuation of the magnitude of the true signal, which is called leakage bias (Swenson and Wahr 2002; Chen et al. 2005).

**Fig. 1:**
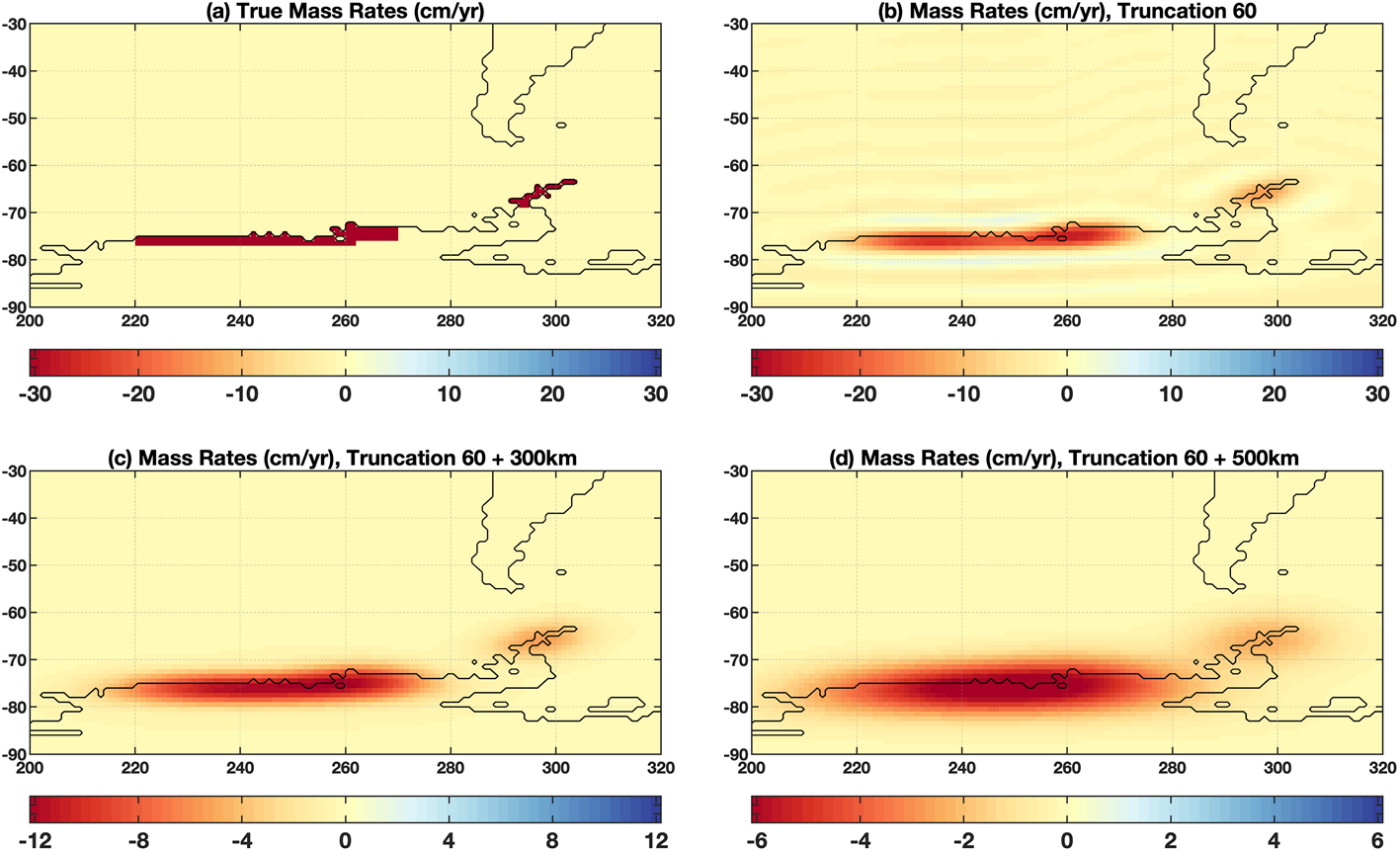
Illustration of leakage and attenuation effects in GRACE/GRACE-FO derived mass change using synthetic mass rates (in cm/year of equivalent water height) over the Antarctic Peninsula and West Antarctica. **a** the synthetic mass rate model with uniform distribution of ice loss of 30 Gigatonne per year (Gt/year) over the Northern Peninsula, and 120 Gt/year over Amundsen Sea Embayment (ASE) coastal regions, corresponding to ~ − 30 cm/year mass rates in the two modeled (blue) regions; **b** mass rates as they would appear when the gravity SH coefficients are truncated at degree and order 60 (with no filtering or smoothing applied); **c** similar to (**b**), but with 300 km Gaussian smoothing applied; **d** similar to (**b**), but with 500 km Gaussian smoothing applied. Different color scales are purposely chosen for the 4 panels to better illustrate the effect (see Chen et al. 2015 for details of the simulations)

To illustrate this challenge, Fig. 1 shows the comparisons of four mass fields (with different smoothing schemes) using experiments based on synthetic data model over West Antarctica (see Chen et al. 2015 for details). In this particular case, the degree-60 truncation of gravity SH coefficients alone would reduce the magnitudes of the ice loss signals (e.g., in the centers of the two modeled areas) by about half. With 300 km Gaussian smoothing (on top of degree-60 truncation), the signals were further attenuated by as much as ~ 80% for over Antarctic Peninsula (e.g., − 30 vs. − 6 cm/year). Therefore, without other means to correct the leakage bias, GRACE/GRACE-FO time-variable gravity solutions will not provide any meaningful mass change estimates at small basin or regional scales. The actual leakage effect of GRACE/GRACE-FO mass fields depends on the spatial scales and distribution of the mass change signals and on the particular filter used.

Reducing leakage bias plays a critical role in quantifying mass variations at basin and regional (and even continental) scales using GRACE/GRACE-FO time-variable gravity solutions. Quantification of GRACE/GRACE-FO leakage bias needs additional information from other independent sources. One commonly used method to correct GRACE/GRACE-FO leakage bias is to use model-derived scale factors (Landerer and Swenson 2012). Land Surface Models (LSMs) can simulate TWS changes at much higher spatial resolution. Despite the expected large uncertainty of LSM TWS estimates, if we assume that GRACE/GRACE-FO and LSM estimates share similar temporal and spatial spectra of TWS changes over land, we can use LSM TWS grids as the synthetic model to carry out similar simulations as illustrated in Fig. 1. Scale factors are derived as the ratios between amplitudes of the original “true” signal and the truncated and filtered signal (e.g., Figs. 1a vs. 1c) at either grid or basin scales. The model-derived scale factors can be applied to GRACE/GRACE-FO estimated mass changes (with similar truncation and spatial filtering applied) to help correct the leakage bias. Similar scale factors can be also derived for polar ice sheets and mountain glaciers mass change, as long as we can construct a reasonable high-resolution mass model from other measurements or model predictions. To overcome the model dependency of the scale factor method, Vishwakarma et al. (2017) explored a data-driven method to reduce leakage error at catchment basin scales, and validated the method using GRACE-type closed-loop simulations. The results show that this improved data-driven method outperforms other methods in 22 out of 32 selected catchment basins of different sizes and located in different climate zones. A recent study (Dobslaw et al. 2020) applied a similar method to approximate leakage effect using the differences of two differently filtered gravity fields to derive a globally constant and time-invariant scale factor that can help correct the leakage bias.

The leakage bias can also be quantified by integrating GRACE/GRACE-FO observations with independently determined source location information through forward modeling (FM). FM was first developed for estimating regional ice loss rates of the Greenland Ice Sheet (GrIS) and Antarctic Ice Sheet (AIS) (Chen et al. 2006a, b). In these cases, the sources and locations of the mass losses are reasonably known. GRACE could easily capture the signals, but with significantly attenuated amplitudes (just like in the experiments shown in Fig. 1). The FM method was designed to reconstruct the “true” magnitude of the mass change through iterative numerical simulations. The simulations start from an a priori mass model that can be arbitrarily defined or simply set to zero. After the same truncation and spatial filtering (applied to GRACE data) are applied to the a priori mass model, the resulted field is compared with GRACE observation (after truncation and spatial filtering), and the difference between the two fields is added back to the a priori model, and then repeat the simulation until the difference reaches a predefined threshold. The process has been proved to converge. Details of the FM method and simulation procedures can be found in Chen et al. (2015). The FM method is also widely used in studies of ice-mass change of mountain glaciers (Chen et al. 2007a, b; Wouters et al. 2008), global mean ocean mass change (Chen et al. 2013; Yi et al. 2015; Jeon et al. 2018), and large lake water storage change (Ni et al. 2017; Chen et al. 2017).

#### Low-Degree SH Coefficients and Geocenter

The lowest degree SH coefficients of GRACE/GRACE-FO gravity solutions, in particular the degree-2 zonal term Δ*C*_2,0_ show substantially larger uncertainty (likely due to a heating issue of ACC). For the late stage of GRACE and the GRACE-FO period, Δ*C*_3,0_ solutions are also subject to relatively larger uncertainty. To improve GRACE/GRACE-FO mass variations of the longest wavelength, independent Δ*C*_2,0_ and Δ*C*_3,0_ solutions observed by Satellite Laser Ranging (SLR) are provided by the GRACE/GRACE-FO SDS (GRACE Technical Note 14), as recommended replacements of GRACE/GRACE-FO solutions (Loomis et al. 2019). SLR has been a proven space-geodetic technique for monitoring low degree gravitational changes (Yoder et al. 1983; Cox and Chao 1998; Cheng and Tapley 1999), and SLR Δ*C*_2,0_ and Δ*C*_3,0_ solutions are generally believed to be more reliable than those from GRACE/GRACE-FO. However, due to limited number of SLR satellites sampling only a few orbit inclinations, SLR *C*_2,0_ and *C*_3,0_ solutions are expected to be affected by difficulties in separating contributions from different zonal terms. This is mitigated by Loomis et al. (2019) in part by forward-modeling the higher-degree zonals based on GRACE results which significantly has improved the estimates of both *C*_2,0_ and *C*_3,0_.

The degree-1 SH coefficients ($\Delta C_{1,1}$,$\Delta S_{1,1}$, and $\Delta C_{1,0}$) are linearly related to the three components of geocenter motion, defined as variations of CM with respect to the center of figure (CF) via the following equations (Heiskanen and Moritz 1967; Crétaux et al. 2002; Swenson et al. 2008),

7 $$\begin{array}{*{20}c} {\Delta C_{1,1} = \frac{1}{\sqrt 3 a}\Delta X_{{{\text{gc}}}} } \\ {\Delta S_{1,1} = \frac{1}{\sqrt 3 a}\Delta Y_{{{\text{gc}}}} } \\ {\Delta C_{1,0} = \frac{1}{\sqrt 3 a}\Delta Z_{{{\text{gc}}}} } \\ \end{array}$$

These degree-1 coefficients are set to zero in GRACE/GRACE-FO gravity solutions, as the gravity field is defined in the CM reference frame. When we estimate mass variations or redistributions in the Earth system using GRACE/GRACE-FO gravity measurements, the mass changes are actually defined in the CF reference frame (e.g., water mass moves from land to ocean, or redistributes among different regions on land). These mass redistributions do not change the CM in the inertial space, but will affect the position of the CF with respect to the CM, or vice versa. Therefore, independently determined geocenter motion or degree-1 SH coefficients ($\Delta C_{1,1}$, $\Delta S_{1,1}$, and $\Delta C_{1,0}$ are needed to complement the GRACE/GRACE-FO time-variable gravity solutions (now in the CF frame). Geocenter motion is expected to mainly affect GRACE/GRACE-FO global and large basin or regional mass change estimates, as the degree-1 SH coefficients represent the longest wavelength mass change in the Earth system. The impact on small basin or regional mass changes should be minimal or negligible.

There are several methods to estimate geocenter motion. One is to use space-geodetic techniques, e.g., observations from SLR, DORIS (Doppler Orbitography and Radio Positioning Integrated by Satellites), and GNSS (Global Navigation Satellite System). SLR is regarded as the most suitable single technique for geocenter variation determination. Accurate quantification of geocenter motion using these geodetic techniques is difficult due to various limitations of these techniques (Wu et al. 2012). Combining GNSS observed land surface deformation with model predicted ocean bottom pressure (OBP) offers another means to solve geocenter motion (Wu et al. 2017). Although GRACE/GRACE-FO gravity solutions do not provide geocenter terms, geocenter motion can be estimated by using GRACE mass change observations over land, combined with model predicted OBP over the ocean (Swenson et al. 2008). This method was later improved by combining GRACE data over land and barystatic sea level change constrained by sea level equation (Sun et al. 2016; Jeon et al. 2018). The updated method leads to significantly improved geocenter motion determinations at long-term time scales. For the GRACE/GRACE-FO SDS RL06 gravity solutions, geocenter motion series are estimated by the SDS using the improved method (Sun et al. 2016), and provided as supplementary datasets (GRACE Technical Note 13, Landerer 2019).

#### Independent Validations of GRACE/GRACE-FO Observations

Another major challenge is how to validate GRACE/GRACE-FO observed time-variable gravity solutions and mass changes at different spatial scales. The difficulty is mainly due to the lack of independent measurements of mass (or gravity) changes that are comparable to GRACE spatial resolution. Low-degree gravitational changes (up to degree and order 5) can be derived from SLR tracking data of multi-satellites, including LAGEOS‐1, LAGEOS‐2 and Starlette, Stella and Ajisai (Cheng et al. 2011). These independently determined low-degree SH coefficients offer important validations of GRACE/GRACE-FO gravity solutions at the longest wavelengths. The two degree-2 order-1 SH coefficients ($\Delta C_{2,1}$ and $\Delta S_{2,1}$) are linearly related to polar motion (PM), the equatorial components of Earth rotational axis (Eubanks 1993; Gross 2007). Polar motion (PM) is regarded as one of the most accurately measured geodetic quantities, thanks to advancements of modern space geodetic techniques. Therefore, PM-derived $\Delta C_{2,1}$ and $\Delta S_{2,1}$ are believed to be more accurate than GRACE/GRACE-FO and SLR observations, especially at long time scales, and can be used to validate GRACE/GRACE-FO solutions [see example applications in Chen et al. (2016) and Göttl et al. (2018)].

Satellite altimetry has been a well-established technique for accurately measuring global sea level change since 1992 (Ablain et al. 2015; Nerem et al. 2018). Altimeter-observed global mean sea level (GMSL) change is driven by two major contributions, barystatic sea level change due to water mass redistribution between the oceans and land (including polar ice sheets), and ocean volume or density change (steric change) due to temperature and salinity variations. The difference between satellite altimeter GMSL and Argo derived steric change provides an independent estimate of barystatic sea level change, and a unique validation of GRACE/GRACE-FO observed mass change on global scale (barystatic sea level represents the largest scale mass change signal in the climate system). Accurate quantification of barystatic sea level from altimeter and Argo data is also challenging, and the uncertainty is mainly from Argo ocean temperature and salinity data due to limited spatial coverage in coastal and high latitude regions and the lack of observations in deep ocean (below 2000 m). Chen et al. (2013) compared GRACE and Argo estimated GMSL changes with altimeter observations over the period 2005–2011, and found remarkable agreements at both seasonal and long-term time scales (e.g., the GRACE + Argo and altimeter GMSL rates are 2.40 ± 0.54 vs. 2.39 ± 0.48 mm/year). The good agreements between GRACE + Argo and altimeter estimates were confirmed by several other studies (Yi et al. 2015; Chambers et al. 2017; Dieng et al. 2017; WCRP 2018). The closure of the GMSL budget is strongly dependent on the choice of GIA corrections (Uebbing et al. 2019).

At regional scales, validation of GRACE/GRACE-FO observed mass (or gravity) changes is even more difficult. In some regions of the world (e.g., the Central Valley and High Plain Aquifer in the US and Murray-Darling Basin in Australia), in situ groundwater level observations from dense well networks are available. These in situ groundwater level observations can be useful for validating GRACE/GRACE-FO observed TWS change, if we can separately estimate water storage changes in surface components of the terrestrial water cycle (i.e., lake, snow, and soil moisture) from other sources (i.e., models) and remove them from GRACE/GRACE-FO observations. Over the past decade, GRACE/GRACE-FO time-variable gravity measurements have captured some significant groundwater depletions in different regions of the world, including Northwest India (NWI), Central Valley (CV) in the US, and North China Plain (NCP). Cross comparisons between GRACE/GRACE-FO gravity and in situ well observations can help validate each other at regional scales. Scanlon et al. (2012) combined GRACE observed TWS change with LSM soil moisture and snow water estimates to study groundwater storage change in the California CV, and found significant groundwater depletion (totaling 31.0 ± 3.0 km^3^) during the period October 2006–March 2010. GRACE estimated CV groundwater depletion agreed with those from in situ well data. Feng et al. (2013) carried out a similar comparison of groundwater depletion in NCP, and also showed good agreement between GRACE-based estimates and well data.

In addition, the Caspian Sea level change offers a unique opportunity for validating GRACE/GRACE-FO gravity solutions at a broad band of frequencies at regional scales (Swenson and Wahr 2007; Chen et al. 2017). The large magnitude and the spatial scale of the Caspian Sea level change within a well-defined geographical location in an arid continental region make its change an ideal signal for validating GRACE observations at regional scales. The Caspian Sea level change is thought to be dominated by water mass change, with a minor steric contribution. As the largest enclosed inland body of water on Earth with a surface area of ~ 371,000 km^2^, the Caspian Sea has undergone substantial fluctuations during the past several hundred years. Driven by imbalanced water fluxes in the Caspian Sea drainage basin, over the GRACE/GRACE-FO period, the Caspian Sea level is dropping at a substantial rate up to − 9 cm/year (for periods after 2005), and the amplitudes of seasonal oscillations of Caspian Sea level change can reach to ~ 20 cm (Chen et al. 2017). To validate GRACE/GRACE-FO estimates at regional scales, a particular challenge is to address GRACE/GRACE-FO leakage bias. The leakage correction methods discussed above (see Sect. 2.3.1) have proven to be helpful. As demonstrated in Chen et al. (2017), after the leakage corrections, GRACE estimated Caspian Sea level change agrees well with independent altimeter observations (e.g., the linear Caspian Sea level trends from GRACE and altimeter are 6.00 ± 0.39 and 6.07 ± 0.26 cm/year, respectively, for the period April 2002–April 2015).

As another independent method to evaluate the quality of time-variable gravity field solutions, orbit tests using ESA’s GOCE mission are used (Dahle et al. 2019; Jäggi et al. 2020). Due to the very low altitude (~ 255 km) of this satellite, its sensitivity to the Earth’s gravity field is rather high. The monthly gravity fields to be validated are used as part of the background modeling in a purely dynamic determination of GOCE orbits which are fitted to kinematic orbit positions used as pseudo-observations. Looking at the resulting orbital fit RMS values for different monthly gravity field solutions provides a valuable metric to compare the different solutions in a relative sense or, when comparing with the fit RMS of a high-resolution static gravity field model, even to assess the absolute accuracy of a time-variable gravity field.

#### Uncertainty Assessment

Quantification of the uncertainty of GRACE/GRACE-FO-estimated mass change is difficult due to the lack of adequate independent observations at scales comparable to the GRACE/GRACE-FO observations. The formal errors provided along with GRACE/GRACE-FO GSM SH coefficients appear too optimistic for quantifying the real uncertainty of GRACE/GRACE-FO mass change estimates, as errors of the background geophysical models and processing methods are mostly unknown. The characterization of errors and error correlations of GRACE gravity solutions has improved significantly over the years (Kvas et al. 2019). A limited number of available other independent datasets are useful for validating GRACE/GRACE-FO observations (see Sect. 2.3.3), but not accurate enough to provide an accurate quantitative assessment of GRACE/GRACE-FO uncertainty level. Differences between similar GRACE/GRACE-FO gravity solutions from different processing centers are evident, and offer a means for evaluating internal consistency among the different solutions. But that is certainly not an ideal or accurate measure of GRACE/GRACE-FO’s real uncertainty, because those solutions are clearly not independent to each other, due to the use of the same background geophysical models and similar data processing methods.

Considering atmospheric and dynamic oceanic mass variations have been largely removed from GRACE/GRACE-FO gravity solutions, the residual signal over the ocean represents only barystatic sea level change (i.e., sea level change introduced by mass exchange between land and ocean), errors of the atmospheric and oceanic models used in GRACE data processing, plus other noise. Barystatic sea level change is mostly governed by the equilibrium ocean surface. The average annual amplitude of barystatic sea level change is ~ 10 mm, with a long-term trend of ~ 2 mm/yearover the GRACE/GRACE-FO time span (Chen et al. 2020; Dieng et al. 2017). If we remove the seasonal and long-term signals in GRACE/GRACE-FO mass fields, the residuals over the open oceans would be a representative measure of noise in GRACE/GRACE-FO mass fields over land, assuming that the noise levels over land and ocean are similar. This provides a means to indirectly quantify the uncertainty of GRACE TWS (and ice mass) change estimates.

**Fig. 2:**
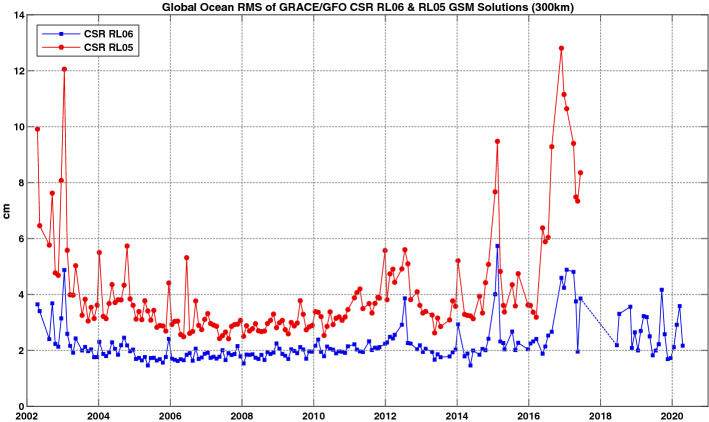
Monthly RMS of GRACE/GRACE-FO global ocean mass change residuals (in cm of equivalent water height) estimated from the CSR RL06 GSM gravity solutions over the period April 2002 and April 2020 (without SLR $\Delta C_{2,0 }$/$\Delta C_{3,0 }$ replacements). The RMS are computed over the open ocean using a 500 km ocean basin kernel and with 300 km Gaussian smoothing. Similar RMS for CSR RL05 GSM solutions are also provided for comparisons

Figure 2 shows an example of estimating the global ocean mean RMS of GRACE/GRACE-FO CSR RL06 solutions over the period April 2002 till April 2020, covering the GRACE period (April 2002 till June 2017) and the first two years of GRACE-FO (June 2018 till April 2020). The GRACE/GRACE-FO mass fields are computed from CSR RL06 GSM solutions with 300 km Gaussian smoothing and without considering the geocenter terms and SLR $\Delta C_{2,0 }$/$\Delta C_{3,0 }$ replacements. For each monthly solution, the global ocean mean RMS was computed over the open ocean using an ocean basin kernel that excludes a 500 km buffer zone along the coasts. Similar ocean RMS for CSR RL05 GSM solutions are also provided for comparison. Before computing the ocean RMS, seasonal and long-term signals are first removed from the ocean mass residuals at each grid point.

The RL06 solutions show significantly smaller RMS than those from RL05 (over the GRACE period), benefitting from improved geophysical background models and data processing procedures. For most part of the GRACE period, the RL06 RMS are ~ 2 cm, which can be taken as an approximate mean RMS level for GRACE RL06 mass change estimates. GRACE-FO appears to show a generally comparable, but slightly higher RMS level compared to GRACE. The relatively higher RMS of the late stage of GRACE and GRACE-FO are likely related to the single accelerometer operating modes of both missions. The accelerometer on one GRACE satellite failed during the late stage of the mission and the same issue happened on GRACE-FO upon launch, and result substantially large noise in the low-degree SH coefficients (in particular $\Delta C_{2,0 }$ and $\Delta C_{3,0 }$). It is worth noting that the open ocean RMS analysis only provides a pointwise assessment of GRACE/GRACE-FO noise level. It involves many other factors when assessing the uncertainty of GRACE/GRACE-FO observed mass change time series averaged over a given region (Groh et al. 2019).

A previous study (Chen et al. 2016) used the Three-Cornered Hat (TCH) method to evaluate the uncertainties of six different estimates of degree-2 gravitational changes ($\Delta C_{2,1}$, $\Delta S_{2,1}$ and $\Delta C_{2,0}$) from Earth rotation, SLR, model prediction, and three GRACE RL05 solutions. The TCH method is a useful tool for quantifying the uncertainty of each individual series when a number of different estimates of the same variable are available, with the assumption that the different series contain the same signal but independent noise (Tavella and Premoli 1993). This can be expressed as,

8 $$\left[ {\begin{array}{*{20}l} {X_{1} \left( t \right) = Y\left( t \right) + \sigma_{1} \left( t \right)} \hfill \\ {X_{2} \left( t \right) = Y\left( t \right) + \sigma_{2} \left( t \right)} \hfill \\ {X_{3} \left( t \right) = Y\left( t \right) + \sigma_{3} \left( t \right)} \hfill \\ \ldots \hfill \\ {X_{n} \left( t \right) = Y\left( t \right) + \sigma_{n} \left( t \right)} \hfill \\ \end{array} } \right.$$

Under the independent noise assumption, the variance of the difference of any two of the estimates can be expressed as,

9 $${\text{var}}\left( {\sigma_{i} } \right) + {\text{var}}\left( {\sigma_{j} } \right) = {\text{var}}\left( {X_{i} - X_{j} } \right)$$

Given the number of estimates (*n*), we can construct $n \cdot \left( {n - 1} \right)/2$ variance equations, and solve $\sigma_{i}$ using least-squares fit. The TCH method can be readily used to quantify uncertainty levels of GRACE/GRACE-FO estimated mass changes at different spatial scales as well. However, errors in the different GRACE/GRACE-FO solutions are not completely independent due to similar background models and processing methods, and TCH only provides approximate estimates of the uncertainty. Nevertheless, the TCH method can be useful for assessing at least internal or relative accuracy of different solutions of the same variable. For example, the TCH analysis in Chen et al. (2016) indicated that among the three GRACE RL05 solutions, the CSR $\Delta C_{2,1}$ and $\Delta S_{2,1}$ showed the best agreements with Earth rotation, SLR, and model estimates, and among the six solutions, the CSR RL05 and Earth rotation estimates yielded the best agreements (with the lowest RMS residuals and highest correlation coefficients).

#### Separation of Different Geophysical Signals

**Fig. 3:**
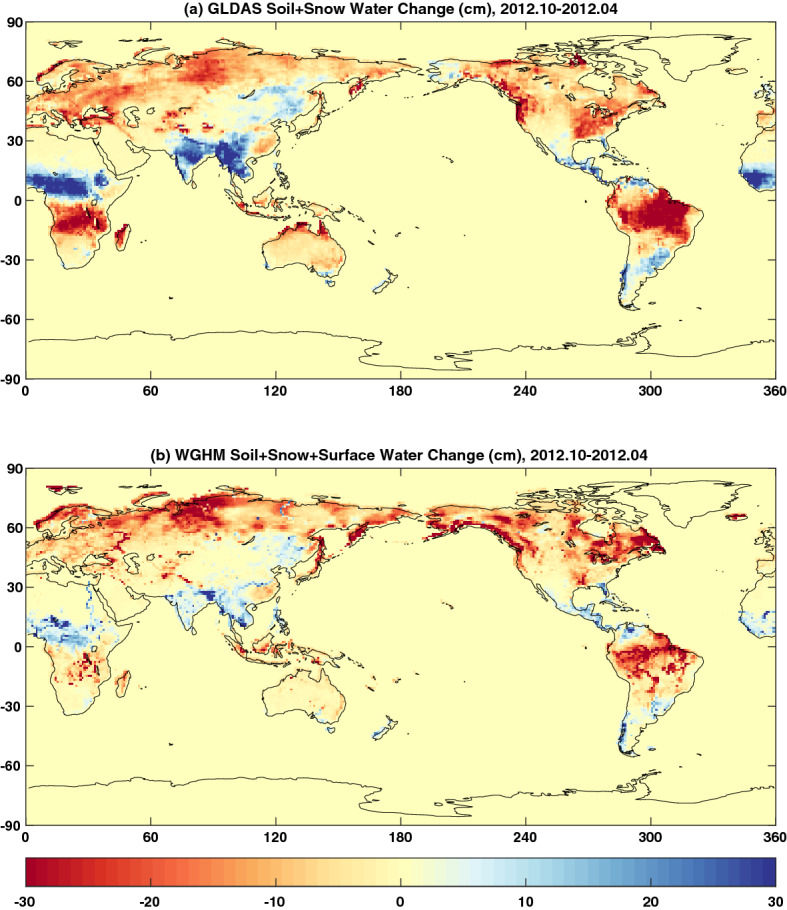
Surface water storage changes (in cm of equivalent water height) between April 2012 and October 2012 (October 2012–April 2012) estimated by **a** the GLDAS Noah and **b** WGHM (version 2.2). Water storage in surface reservoirs is not included in GLDAS Noah

With the 2D assumption, we are able to estimate mass change on the Earth surface using GRACE/GRACE-FO gravity solutions. However, the estimated mass change may involve different contributing sources, e.g., different components of the surface geophysical fluid envelope and mass transport within the solid Earth (e.g., from GIA and earthquakes). Separating the different contributing sources requires independent knowledge from either numerical model predictions or other observational techniques. For example, quantification of groundwater storage change using GRACE/GRACE-FO data relies on the effective removal of surface water storage change using model estimates and/or limited available observations, which largely affect the accuracy of GRACE/GRACE-FO groundwater estimates (Rodell et al. 2009). Different LSMs can lead to significantly large different estimates of water storage changes, due to limitations and immaturity of the models. This is clearly illustrated by the comparisons (in Fig. 3) of global surface water storage changes (i.e., in soil moisture, snow, and surface reservoir) between October 2012 and April 2012 from two commonly used LSMs, the Global Land Data Assimilation System (GLDAS) Noah (Rodell et al. 2004) and the WaterGAP Global Hydrology Model (WGHM) (Döll et al. 2003). At seasonal scales, the differences between the two models can reach to ~ 10–20 cm of equivalent water height (e.g., in South Asia and Africa). Large discrepancies between the models also exit at other time scales. Improved accuracy of model-predicted surface water storage change plays a key role in studying groundwater change using GRACE/GRACE-FO data.

Uncertainty of model predicted GIA deformation is a major error source for GRACE/GRACE-FO estimated mass rates of the Antarctica and Greenland ice sheets and mountain glaciers. GIA is a slow uplift of the solid Earth as a delayed viscoelastic response to mass load removal following the Last Glacial Maximum (Peltier 2004). GIA is often represented by linear deformation of the Earth surface (at hundreds to thousands years time scales). To estimate ice-mass change rate using GRACE/GRACE-FO gravity solutions, GIA effects in the studied region need to be removed from GRACE/GRACE-FO measurements using estimates from models. The uncertainty of model-predicted GIA effects is believed to be the largest error source of Antarctic ice-mass rates estimated by GRACE/GRACE-FO, which can be as large as ± 72 Gt/year (Velicogna and Wahr 2013). Over Greenland, the GIA uncertainty is significantly smaller, ranging from ± 7 to ± 21 Gt/yearbased on different estimations (Barletta et al. 2013; Velicogna and Wahr 2013; IMBIE Team 2020). GIA uncertainty also affects GRACE/GRACE-FO estimates of ice-mass balance of mountain glaciers, although GRACE/GRACE-FO leakage error may play a more significant role for mountain glaciers due to their relatively smaller spatial scales. In addition, accurate quantification of global ocean mass change using GRACE/GRACE-FO measurements also depends on successful removal of solid Earth geophysical signals. The GIA uncertainty in terms of GRACE/GRACE-FO derived global ocean mass rates is estimated ~  ± 0.3 mm/year (Chambers et al. 2010; WCRP Sea Level Budget Group 2018).

#### Other Issues

In addition to the major challenges discussed above, there are many other issues we need to deal with as well when using GRACE/GRACE-FO satellite gravity measurements to study mass variations in the Earth system. As we mentioned above, in GRACE/GRACE-FO GSM gravity solutions, the $\Delta C_{00}$ coefficients are set to zero due to global mass conservation. This mass conservation only applies to the entire Earth system, i.e., the total gravity field including the atmosphere (GSM + GAC), which means that at any given time, the sum of the $\Delta C_{0,0}$ coefficients of the GSM and GAC fields equals to zero ($\Delta C_{0,0}^{{{\text{GSM}}}} + \Delta C_{0,0}^{{{\text{GAC}}}} = 0$). Since atmospheric and dynamic oceanic mass variations (i.e., GAC fields) have been removed from GRACE/GRACE-FO GSM solutions during the data processing by using a de-aliasing model, if the total mass of the GAC fields ($\Delta C_{0,0}^{{{\text{GAC}}}}$) is not conserved, then neither is the total mass of GSM, as

10 $$\Delta C_{0,0}^{{{\text{GSM}}}} = - \Delta C_{0,0}^{{{\text{GAC}}}}$$

For each GSM solution, the corresponding GAC field equals to the sum of atmospheric and dynamic oceanic mass change estimated models. The ocean model estimates used in the RL06 GAC fields are based on the Max-Planck-Institute for Meteorology Ocean Model (MPIOM). MPIOM runs with the Boussinesq approximation to conserve the total water volume. A separate so-called Greatbatch correction was implemented when generating RL06 AOD1B dealiasing products (Dobslaw et al. 2017b). That means the total mass of the ocean part of the GAC field is conserved. However, the total mass of the atmosphere is not constant, which means that $\Delta C_{0,0}^{{{\text{GAC}}}}$ represents variations of global mean atmospheric mass (GMAM). Therefore, to correctly implement global mass conservation, $- \Delta C_{0,0}^{{{\text{GAC}}}}$ needs to be added to GRACE/GRACE-FO GSM solution. Please note $\Delta C_{0,0}^{{{\text{GAC}}}}$ and $\Delta C_{0,0}^{{{\text{GAA}}}}$ should be exactly the same by definition, as GAA fields represent atmospheric mass change.

**Fig. 4:**
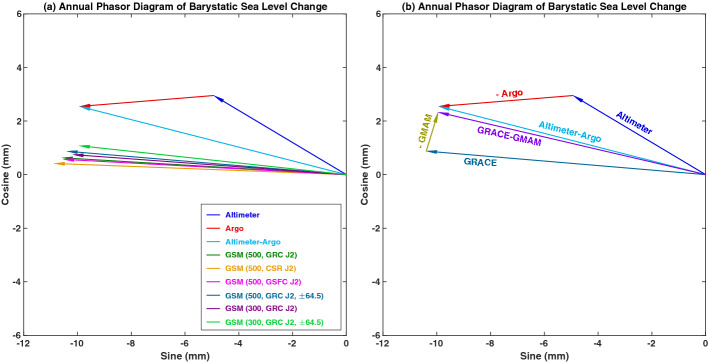
**a** Annual phasor diagram of barystatic sea level changes observed by satellite altimeter and Argo (Altimeter-Argo) and GRACE CSR RL06 GSM solutions over the period January 2005–December 2015. The five GRACE GSM estimates are based on different treatments of $\Delta C_{2,0 }$(noted as J2 in the legend), ocean basin kernel, and latitude ranges of integration (see Chen et al. 2019 for details); **b** Annual phasor diagram of barystatic sea level from Altimeter-Argo and GRACE GSM (500 km, GRC J2, ± 64.5) with $\Delta C_{0,0}^{{{\text{GSM}}}}$ effect considered

The $\Delta C_{0,0}^{{{\text{GSM}}}}$ effect is rather small, but has notable impact on GRACE/GRACE-FO observed global mean ocean mass change, also known as barystatic sea level change (Gregory et al. 2019). Although GRACE/GRACE-FO observed barystatic sea level change generally agrees well with the difference between satellite altimeter sea surface height and Argo steric results, there appear a slight and systematic annual phase lag (of ~ 10° or 10 days) between GRACE/GRACE-FO and altimeter GMSL minus Argo steric effect (see Fig. 4a). This phase lag was found to be attributed to the “missing” of the $\Delta C_{0,0}$ terms in the GSM solutions. When the $\Delta C_{0,0}$ terms are considered (using $- \Delta C_{0,0}^{{{\text{GAC}}}}$ provided in the GRACE/GRACE-FO supplementary GAC fields), the annual phase lag between the two independent estimates is significantly reduced (see Fig. 4b), and the agreement between the two estimates at seasonal time scale is greatly improved. The $\Delta C_{0,0}$ effect on basin and regional TWS and ice-mass changes is clearly negligible. A related analysis was discussed in details in Chen et al. (2019).

A spherical Earth model has been used in most mass inversions from GRACE/GRACE-FO time-variable gravity fields, for the sake of mathematical convenience (Wahr et al. 1998). The ellipsoidal shape of the actual Earth has a small but clearly non-negligible effect on GRACE/GRACE-FO estimated surface mass changes, especially in polar regions and at small basin scales (Li et al. 2017; Ditmar 2018; Ghobadi-Far et al. 2019). The ellipsoidal correction to GRACE observed Greenland ice-mass rate is estimated to be ~ 4% of the signal, and the effect can be up to ~ 7% for smaller regions like Svalbard (Li et al. 2017). On a positive note, the ellipsoidal effect has been considered in the current GRACE/GRACE-FO JPL RL06 and CSR RL06 (v2) mascon solutions (Wiese et al. 2016).

Seismic deformation of large earthquakes may also have a notable effect on GRACE/GRACE-FO estimated mass changes that are related to the climate system. A recent study (Tang et al. 2020) suggests that sea floor deformations due to several large off-shore earthquakes during the GRACE era could affect GRACE-estimated global ocean mass change rate by ~ 0.07 mm/year. The correction is fairly small compared to the GRACE uncertainty level; however, the consideration of this effect is expected to slightly improve GRACE estimates (Tang et al. 2020).

In the following sections, we will discuss applications of GRACE/GRAC-FO in different components of the Earth system, including land hydrology, cryosphere, ocean, solid Earth geophysics, and deep Earth process using some example analyses.

## Terrestrial Water Storage Change from GRACE/GRACE-FO

**Fig. 5:**
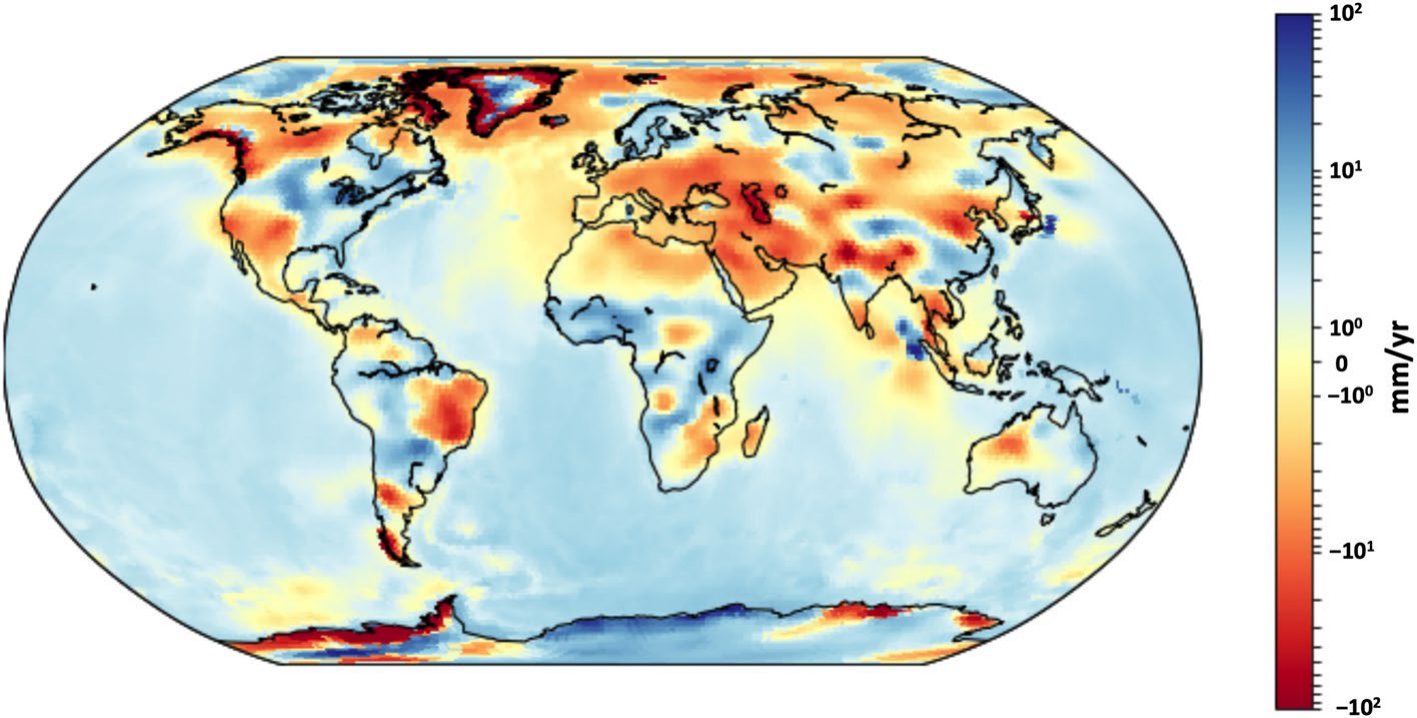
GRACE/GRACE-FO based mass trends calculated from the CSR RL06 mascon solutions over April 2002–August 2020 in mm/year of equivalent water height. The CSR RL06 mascon solutions were corrected for GIA using the ICE6G-D model (Peltier et al. 2018)

The regional mass trends in GRACE/GRACE-FO records are shown in Fig. 5 for the April 2002–August 2020 time period (with GIA removed). Strong positive or negative trends, sometimes exceeding 10 mm/year, are clearly visible over major river basins.

Over river basins, GRACE and GRACE-FO missions measure the vertically integrated water storage change, without separating the contributions from the different components (surface waters, upper few meters of soil and groundwater). This is one fundamental term of the water balance equation relating the derivative of total water storage to precipitation (*P*), evapotranspiration (*E*) and runoff (*R*) over land:

11 $$d{\text{TWS}}\left( t \right)/dt = P\left( t \right) - E\left( t \right) - R\left( t \right)$$

where TWS means vertically integrated water storage, and *t* is time.

No other space technique is able to provide such a quantity of invaluable interest for studying the continental water cycle and quantifying water resources on global scale (e.g., Famiglietti et al. 2015). While global climate and hydrological models can estimate TWS change, in situ networks are unable to provide this information at regional basin scale (e.g., Rodell and Famiglietti 1999, Shiklomanov et al. 2002).

Over 700 articles have been published so far (e.g., ISI Web of Science) since the launch of the GRACE mission on the use of GRACE to estimate temporal changes in land water storage at river basin scale. The recent review by Tapley et al. (2019) summarizes the main contributions of GRACE to land hydrology.

In most river basins, TWS changes observed by GRACE/GRACE-FO are caused by natural climate variability. These changes exhibit complex modes of variability at interannual and decadal timescales, often masking secular trends due to climate change or other geophysical signals. A recent study (Vishwakarma et al. 2021) argued that one should not take GRACE/GRACE-FO trends at face value, but rather relative to the natural variability (trend-to-variability ratio). Emerging trends in TWS indeed show the effect of recent droughts in Europe, Southeast Brazil and Southwestern US. We can also see a progression from dry to wetter conditions in central North America, along the Amazon and Parana rivers in South America, in Southwest Africa, across the Zambezi basin and in Northeast Australia. Despite the large influence of natural climate variability, we can also see anthropogenic influences emerging in TWS changes, often due to groundwater extraction, such as in NWI, NCP, CV in California, and Middle East. In most cases, anthropogenic drivers are convoluted with the natural variability of TWS, making it particularly difficult to assess the impact of climate change on freshwater resources (Asoka and Mishra 2020).

**Fig. 6:**
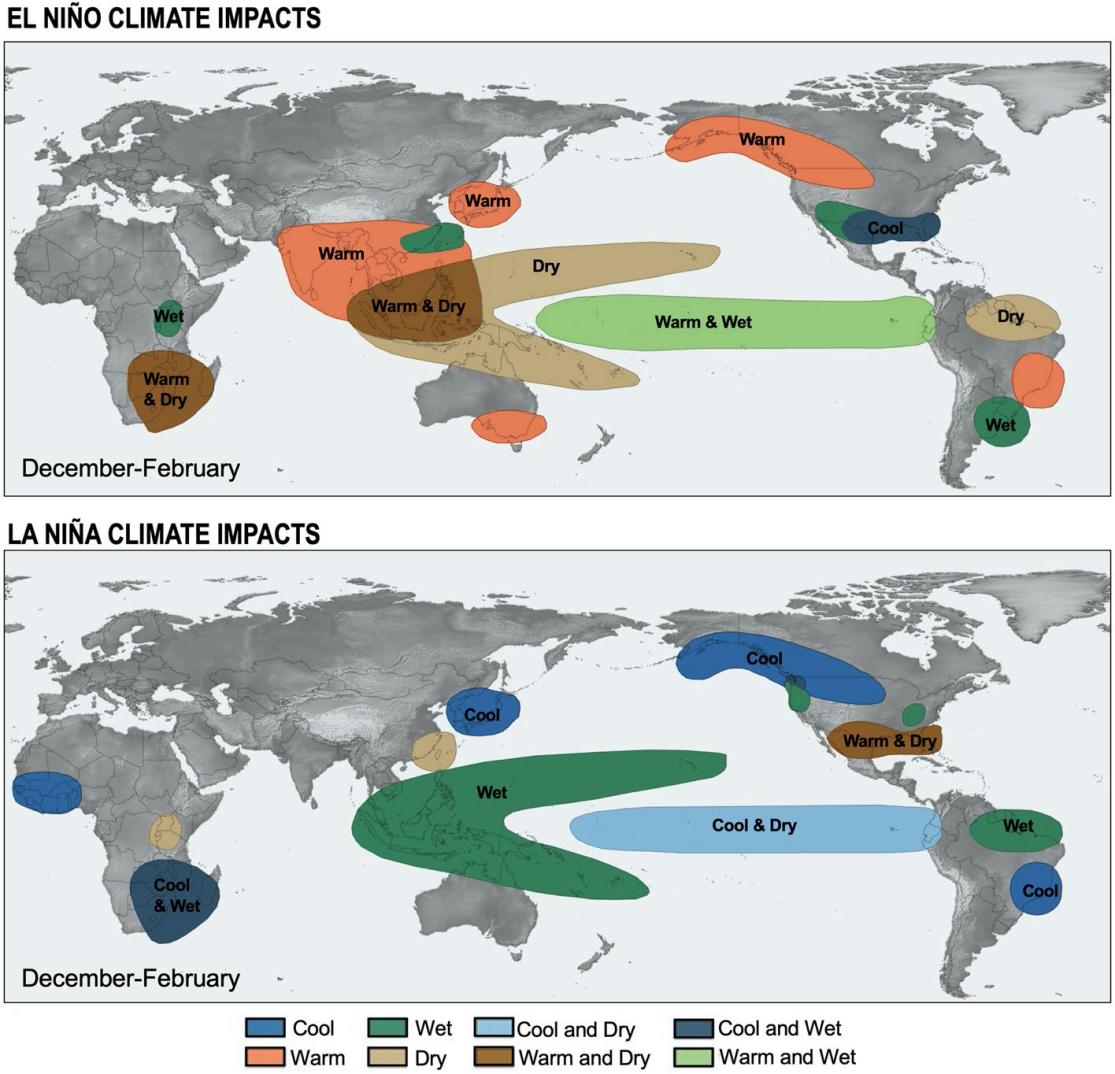
Hydro-meteorological impacts felt worldwide during El Niño (top panel) and La Niña (bottom panel) events (courtesy of World Meteorological Organization)

At interannual time scales, TWS change is mostly driven by natural climate modes, such as ENSO (El Niño Southern Oscillation), PDO (Pacific Decadal Oscillation), NAO (North Atlantic Oscillation) and AMO (Atlantic Multidecadal Oscillation) (Fasullo et al. 2016; Ni et al. 2018; Pfeffer et al. 2021). ENSO is the dominant mode of internal/natural variability of the climate system. Changes in weather systems during both warm (El Niño) and cold (La Niña) events give rise to extreme events (Cai et al. 2015). Among these, severe floods and droughts lasting several months, regularly cause highly negative impacts on human societies and economy (Ropelowski and Halpert 1987). Figure 6 illustrates the regions affected by extreme hydro-meteorological impacts during El Niño and La Niña events.

**Fig. 7:**
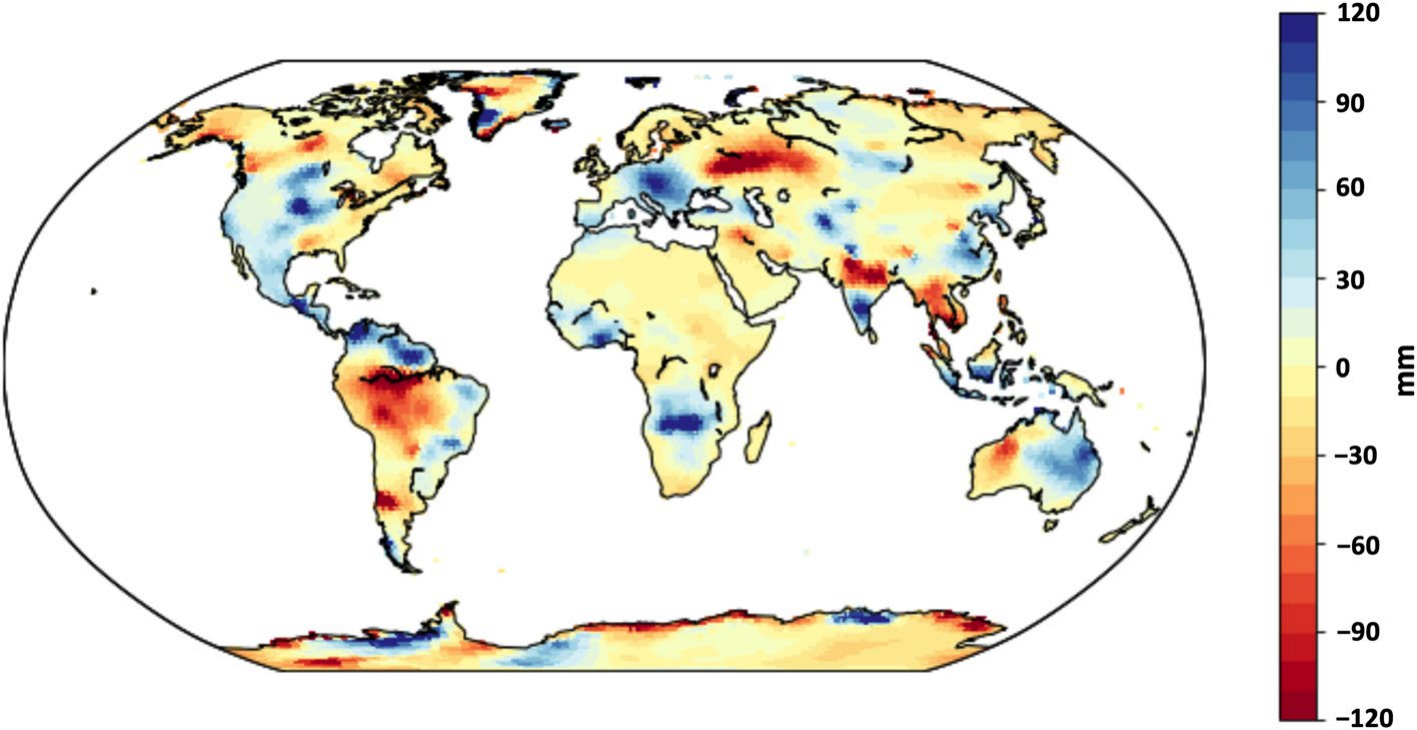
Mean terrestrial water storage anomaly (in mm equivalent water height) during the 2010–2011 La Niña (average over June 2010 and February 2011) calculated from detrended and deseasoned CSR RL06 mascon solutions. Blue (red) colors correspond to an excess (a deficit) in terrestrial water storage with respect to the mean for the January 2003–December 2015 time period

During El Niño phases, there is a precipitation excess over the tropical Pacific and a precipitation deficit across tropical continental areas (Dai and Wigley 2000; Gu and Adler 2011). The inverse is observed during La Niña phases, although the continental areas impacted are not necessarily the same. Here we provide a few examples of impacts on land hydrology during recent ENSO events based on GRACE data. Figure 7 shows the GRACE-based TWS during the 2011 La Niña. The map clearly shows the water excess over the eastern part of Australia during that period.

Boening et al. (2012) and Fasullo et al. (2013) reported strong precipitation over the Australian continent during the 2010–2011 La Niña. They highlighted wetter than normal conditions in various regions of the world, in particular in northern Australia. During an El Niño, other tropical basins are affected by droughts. For example, the 2015–2016 El Niño led to one of the most intense droughts ever recorded over southern Africa (e.g., Siderius et al. 2018). This event prevented groundwater from recharge, hence produced groundwater decline over two consecutive years as reported from an analysis of GRACE data (Kolosu et al. 2019). The extreme drought affecting the Amazon basin in 2005 was also reported in satellite altimetry (Pfeffer et al. 2014) and GRACE observations (Chen et al. 2009; Frappart et al. 2019) and attributed to rainfall deficit in prior years in connection with the 2002–2003 El Niño event. Many other studies have been published on the influence of ENSO events on flood risk and droughts around the world (e.g., Ward et al. 2014; Fok et al. 2018).

While it is the most widely studied example, the impact of climate modes on the water storage variability is not limited to ENSO. The PDO and AMO are two major drivers of the climate system, with large impacts on the terrestrial water storage reported in South America (Ndehedehe and Ferreira, 2019), North America (Kuss and Gurdak 2014) and West Africa (Ndehedehe et al. 2017). The Arctic Oscillation (AO), sometimes associated with NAO, was found to impact the water mass redistribution measured by GRACE in the Northern hemisphere (Matsuo and Heki, 2012). More and more often, it is found that a combination of climate modes is necessary to better represent complex and ephemeral climate conditions, impacting the wind regime, temperature and precipitations, hence TWS (e.g., Xie et al. 2019; Kundzewicz et al. 2019). Due to the global warming of the Earth’s climate, the influences of ENSO (Cai et al. 2015) and other modes such as AMO (Barichivich et al. 2018) are expected to grow and intensify, leading to more frequent and more severe extreme events (e.g., droughts, floods, cyclones, and wildfires). More frequent and larger extremes are thus to be expected in the integrated TWS as well, making the GRACE-FO and future gravity missions critical observation systems, needed to monitor and predict the evolution of water resources under global climate change.

GRACE is also unique in providing information on aquifers depletion due to groundwater pumping for crop irrigation and domestic water use. Several studies have highlighted the importance of GRACE to monitor groundwater resources, in particular some significant groundwater depletions in several large aquifers around the world due to human activities (e.g., Rodell et al. 2009; Tiwari et al. 2009, Famiglietti et al. 2011, Tapley et al. 2019).

Changes in TWS are routinely estimated by global hydrological models that compute water and energy balances at the Earth surface, in response to prescribed atmospheric data (temperature, humidity and wind) and the incident water and energy fluxes from the atmosphere (precipitation and radiation). Meteorological forcing, usually based on atmospheric model reanalyses, represents the largest source of uncertainties in model-based TWS estimations (e.g., Beck et al. 2017; Schellekens et al. 2017). Another source of uncertainty is the treatment of subsurface storage in soils and aquifers, as well as dynamic changes in storage capacity. The study by Scanlon et al. (2018) compared water storage trends from two global hydrological models to GRACE storage trends, and found that models estimated the opposite trend in net land water storage to GRACE over the 2002–2014 period. They attributed this discrepancy to model deficiencies, in particular soil depth limitations.

However, in order to remove the TWS signal over continental areas in GRACE observations, for detecting solid Earth geophysical signals, independent information on TWS will be needed, and this will mostly be based on the use of global hydrological model outputs. An increased accuracy in the GRACE-FO measurements, extended length of the satellite gravity record and expected improvements in modeling should nevertheless help separating hydrological from geophysical signals in order to unveil low amplitude signals originating from the deep Earth interior.

## Mass Change of Polar Ice Sheets and Glaciers from GRACE/GRACE-FO

Mass changes of the polar ice sheets and mountain glaciers have become more and more of scientific as well as societal interest during the past decades since global warming has led to an increased melting of ice masses, which is a major driver of global mean sea level rise and an indicator of climate change worldwide. Several geodetic techniques provide ice sheet mass balance estimates, including satellite altimetry, space-borne interferometric synthetic aperture radar (InSAR), and satellite gravimetry (Shepherd et al. 2012). The latter is the only technique which directly observes mass change from space. Furthermore, with its temporal resolution of typically one month, GRACE does not only provide information about long-term trends but also measures seasonal mass fluctuations. From shortly after release of the first time-variable GRACE gravity field solutions until present, a large number of studies related to ice-mass changes have been published. With increasing length of the time series and improved quality of the solutions, mass trend estimates became more and more robust and accurate. A broad overview of cryospheric applications and important scientific studies is provided by, e.g., Tapley et al. (2019) and Chen (2019); however, they naturally include publications where the use of satellite gravimetry is limited to a maximum time span from 2002 till 2017, i.e., only GRACE data is used. In the following, the focus is on summarizing recent results and studies which also include data of the GRACE-FO mission.

When first analyzing GRACE and GRACE-FO data together as one single gravimetry time series, a key challenge was to properly evaluate whether there are any potential biases or discontinuities between the two missions. As there is no temporal overlap between GRACE and GRACE-FO, this question cannot be answered by a direct comparison but requires independent data sets (e.g., Yi and Sneeuw 2021). By assessing the difference between surface mass balance from regional climate models and ice discharge into the ocean, Velicogna et al. (2020) demonstrate data continuity for the GRACE and GRACE-FO missions over the GrIS and AIS at both the continental and regional scales. For the GrIS, Sasgen et al. (2020a) confirm these findings using a similar approach. For glaciers and ice caps outside Greenland and Antarctica, Ciraci et al. (2020) use atmospheric reanalysis data to also confirm consistency of the two missions.

Already about 3 years after launch, GRACE-FO has justified the strong request by the scientific user community to prolong the GRACE gravimetry time series. Recent results from Sasgen et al. (2020a) show that large year-to-year variability over the GrIS makes it crucial to have continuous satellite observations: while estimating an average ice-mass loss of − 235 ± 29 Gt/year in the period from January 2003 till December 2018, they report that during 2017 and 2018 mass loss rates reached their minimum during this period, just to be followed by the largest annual mass loss recorded since mid of the past century with − 532 ± 58 Gt/year in 2019. For the slightly longer period from April 2002 till September 2019, Velicogna et al. (2020) estimate the average mass loss for Greenland to be − 261 ± 45 Gt/year, while for the AIS they report − 104 ± 57 Gt/year. During the same period, the total average mass loss for glaciers and ice caps amounts to − 281.5 ± 30 Gt/year, dominated mostly by regions in the Arctic (Ciraci et al. 2020). According to these rates, the total mass loss of the polar ice sheets and worldwide glacier systems since the beginning of the GRACE era is equivalent to about 30 mm of global mean sea level rise. It has to be emphasized again that annual mass loss rates strongly depend on the period of considered observations and a longer continuous time series of satellite gravimetry is required, in particular if one aims at separating long-term accelerations in ice-mass loss from short-term ice sheet variability (Wouters et al. 2013).

**Fig. 8:**
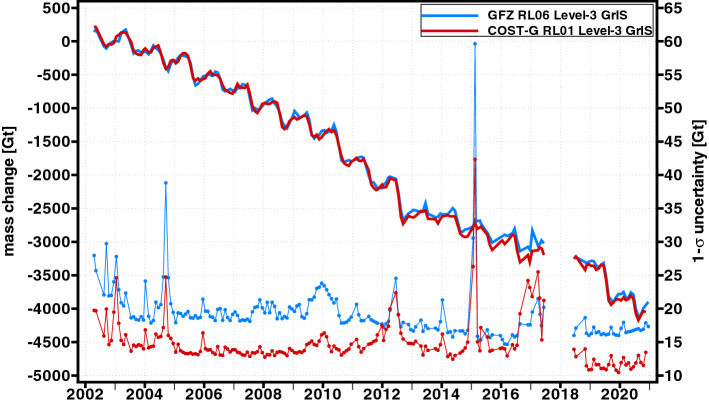
Monthly mass change estimates for the GrIS (solid lines) based on the GFZ RL06 (blue) and COST-G RL01 (red) time series and their associated 1-sigma uncertainties (dotted lines). Shown data are Level-3 ice-mass change products (Sasgen et al. 2019a, 2020b) from GFZ’s GravIS portal (http://gravis.gfz-potsdam.de)

**Fig. 9:**
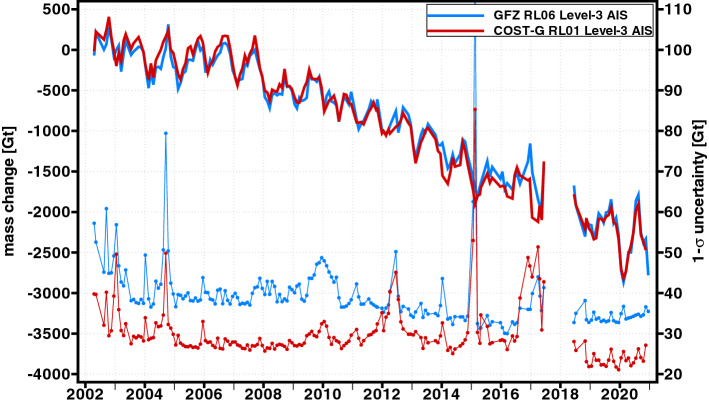
Monthly mass change estimates for the AIS (solid lines) based on the GFZ RL06 (blue) and COST-G RL01 (red) time series and their associated 1-sigma uncertainties (dotted lines). Shown data are Level-3 ice-mass change products (Sasgen et al. 2019a, 2020b) from GFZ’s GravIS portal (http://gravis.gfz-potsdam.de)

Such continuous ice-mass change time series for the GrIS and AIS which are regularly updated on an operational basis are, e.g., provided by GFZ via the Gravity Information Service (GravIS; http://gravis.gfz-potsdam.de) portal. GravIS offers both gridded products as well as regional basin average products of the polar ice sheets based on the GFZ RL06 (Sasgen et al. 2019a) and the COST-G RL01 (Sasgen et al. 2020b) GRACE/GRACE-FO time series. Figure 8 shows that mass change estimates from these two different gravity field time series for the GrIS are generally in good agreement. Unsurprisingly, the COST-G time series which is a combination of various individual time series from different processing centers looks slightly less noisy and its monthly uncertainties are, except for the last few GRACE months, significantly smaller. Visible variations in the uncertainty time series mostly reflect the ground track coverage, i.e., large peaks correlate with known short-period repeat orbit patterns of the satellites. The total ice-mass loss from January 2003 till December 2020 amounts to approximately 4000 Gt which is equal to about 11 mm of sea level rise. Similar conclusions can also be drawn for the AIS (Fig. 9). However, uncertainties are about twice as large as for Greenland, and the mass change time series looks noisier. This is likely because the Antarctica covers a much larger area and different parts of the Antarctica show distinctively different characters in terms of ice-mass changes. Another source is the poorly constrained dealiasing products in this region (Kim et al. 2016; Dobslaw et al. 2017a). Here, the cumulated ice-mass loss from January 2003 till December 2020 adds up to nearly 2500 Gt contributing about 7 mm to the global mean sea level.

As already discussed above, there are several sources of uncertainty in gravimetric mass change estimates. Leakage errors (see Sect. 2.3.1) are generally larger when assessing ice-mass change for smaller sub-regions of the polar ice sheets or mountain glaciers. Besides leakage from surrounding continental masses (i.e., either hydrological or cryospheric leakage), also leakage from sea level change will play a significant role in ice sheet mass balance estimates, especially with further increasing mass losses (Sutterley et al. 2020). A potential way to mitigate leakage errors which are inherently present due to the rather coarse spatial resolution (few hundreds of kilometers) of GRACE/GRACE-FO could also be a combination with satellite altimetry data which offers a much higher spatial resolution (few kilometers). Sasgen et al. (2019b) combined GRACE and CryoSat-2 in the spectral domain to obtain ice-mass balance for the AIS with smaller uncertainties and reduced systematic noise compared to single-sensor analysis. Regarding the not well-determined *C*_2,0_ and (only since November 2016) *C*_3,0_ coefficients from GRACE/GRACE-FO (see Sect. 2.3.2), one can deal with by replacing these coefficients with more robust ones estimated from SLR. Yet it is worth to be mentioned in this section that the *C*_3,0_ coefficient has a large impact particularly on mass balance estimates for Antarctica due to its geographic location (Loomis et al. 2020). The uncertainty of GIA models is another error source which affects ice-mass balance from satellite gravimetry. For the AIS, this is even supposed to be the dominant error source (Velicogna and Wahr 2006; Ivins et al. 2013), whereas GIA model errors are relatively smaller for the GrIS. In an extensive comparative study, The IMBIE Team (2020) shows that estimates of six different GIA models agree well with a standard deviation of ~ 20 Gt/year, which is a rather small fraction of the total signal of ice-mass loss in Greenland.

## Global Mean Oceanic Mass Change from GRACE/GRACE-FO

In this section, we discuss different approaches to estimate the global mean ocean mass change known as barystatic sea level change (Gregory et al. 2019). Mountain glaciers are melting and ice sheets have been losing mass for decades leading to net continental freshwater discharges into the oceans. In addition, TWS changes also influence the decadal variability in ocean mass (Reager et al. 2016).

### Direct Estimate Based on GRACE/GRACE-FO Data

GRACE and GRACE-FO have offered the opportunity to directly estimate the barystatic sea level on a monthly basis. We consider GRACE and GRACE-FO RL06 monthly mean solutions provided by the three data processing centers CSR, JPL and GFZ covering the period from January 2003 to December 2019. Level-3 (L3) ocean mass change fields derived from CSR, JPL and GFZ RL06 gravity spherical harmonic solutions are available through https://podaac-tools.jpl.nasa.gov/drive/files/allData/grace/L3/ocean_mass/RL06/v03 and https://podaac-tools.jpl.nasa.gov/drive/files/allData/gracefo/L3/ocean_mass/RL06/v03 (Landerer 2020a, b, c, d, e, f). We also take advantage of the JPL and CSR RL06 mass concentration (mascon) solutions available via https://podaac.jpl.nasa.gov/dataset/TELLUS_GRAC-GRFO_MASCON_CRI_GRID_RL06_V2 and http://www2.csr.utexas.edu/grace/RL06_mascons.html (date of download: 29.12.2020) (Save et al. 2016, 2020; Wiese et al. 2016). As we are interested in barystatic sea level change, all these solutions were corrected for atmospheric and dynamic ocean effects using the GAD product which contains the monthly mean ocean bottom pressure caused by non-tidal oceanic and atmospheric mass variations. In addition, we have considered the GFZ RL06 ocean bottom pressure L3 product from the GravIS portal (Dobslaw et al. 2019). For this product, individual variables for barystatic sea level variations, residual ocean circulation and modeled oceanic and atmospheric variations are provided. Consequently, we only use the barystatic sea level variable here. Because of the coarse spatial resolution of GRACE and GRACE-FO, continental signal may leak into the coastal areas. To correct this, we follow the procedure described by Chen et al. (2020) based on using an ocean mask with a 500-km buffer zone from the coasts. For the CSR mascon solutions, we considered a 200-km buffer zone (as in Chen et al. 2020), to reduce the relatively smaller but still evident leakage effect in the CSR mascon solutions. The leakage errors are already addressed for the JPL mascon solutions by the Coastal Resolution Improvement (CRI) filter, which is designed to remove leakage between land and ocean signals (Wiese et al. 2016). Prior to spatially averaging the data, we masked out the region in the Eastern Indian Ocean and the Japan coastlines both affected by earthquakes in 2004 and 2011, respectively.

**Fig. 10:**
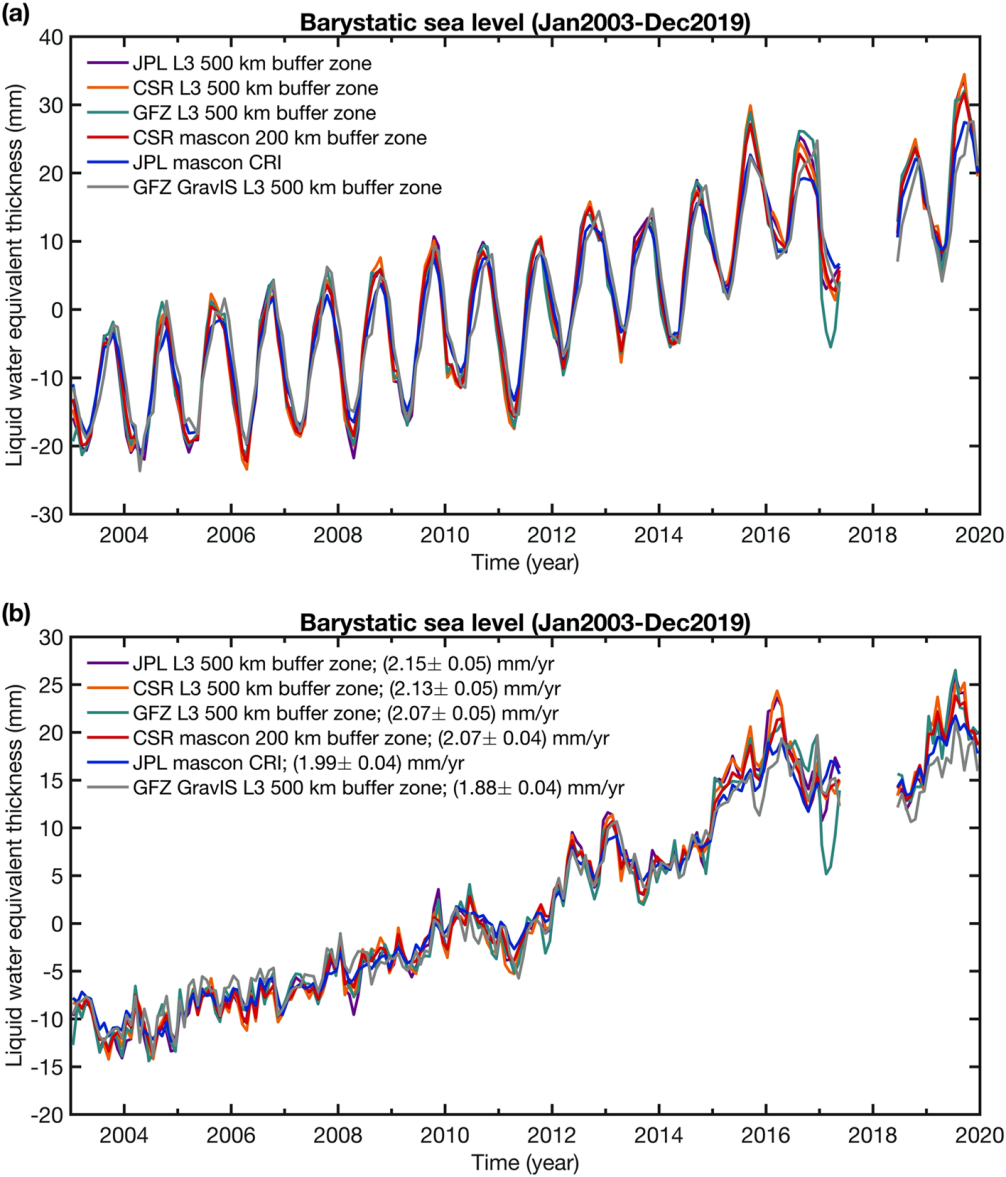
**a** Monthly mean estimates of barystatic sea level change from GRACE/GRACE-FO spherical harmonic Level-3 (L3) fields and mascon solutions over the period January 2003 and December 2019. **b** Same as (**a**) but without the annual signal. Linear trends over 2003–2019 are stated in panel (**b**)

Figure 10a presents the different barystatic sea level time series (in mm). These estimates show a fairly good agreement between each other. The main differences between the solutions appear at the end of the GRACE mission, from half of 2016 and into 2017. This period is known to have degraded data mainly due to problems in the accelerometer instrument, which lead to increased errors in GRACE gravity solutions (Landerer et al. 2020). Figure 10b shows the barystatic sea level corrected for the annual signal. We subtracted the monthly climatological averages to every month to remove the annual signal. These climatological averages are computed only over the GRACE period (over January 2003–December 2016). The estimated linear trends and uncertainties are listed in the legend of Fig. 10b using a least-squares regression model. The actual uncertainties of GRACE/GRACE-FO estimated barystatic sea level rates are expected to be significantly larger due to errors in the GIA correction, geocenter motion contribution, and GRACE/GRACE-FO data. The GIA error alone may contribute ~  ± 0.3 mm/year to GRACE/GRACE-FO barystatic sea level rate uncertainty (Chambers et al. 2010), and the geocenter motion uncertainty may contribute ~  ± 0.21 mm/year (Blazquez et al. 2018). We find positive trends for barystatic sea level ranging from 1.88 to 2.15 mm/year over January 2003 to December 2019. It has to be mentioned that differences in these numbers are not only due to the different GRACE/GRACE-FO time series but might also have their origin in slightly different postprocessing choices applied to the shown barystatic sea level time series (Dobslaw et al. 2020). Yet, our trend estimates are in line with recent literature (Amin et al. 2020; Barnoud et al. 2021) in spite of being estimated over slightly different time periods. Note that the quoted uncertainties only represent the misfit from the linear regression.

### Sea Level Budget Approach

GMSL rises not only because of the increase of freshwater coming from land, but also from ocean volume change due to temperature and salinity variations. This contribution is known as steric sea level. Since 1993, satellite altimetry records a global mean sea level trend of 3.1 mm/year (WCRP Sea Level Budget Group 2018). We considered here the mean estimate from 4 different solutions (CMEMS data available at https://marine.copernicus.eu/, CU from https://sealevel.colorado.edu/, CSIRO downloaded at https://www.cmar.csiro.au/sealevel/sl_data_cmar.html, and JPL data downloaded at https://podaac.jpl.nasa.gov/dataset/SEA_SURFACE_HEIGHT_ALT_GRIDS_L4_2SATS_5DAY_6THDEG_V_JPL1812) to assess the GMSL evolution.

Since the beginning of the 2000s, with the launch of the Argo international program, we have now access to an unprecedented amount of temperature and salinity measurements down to 2000 m depth. Those profiles are most valuable to directly assess the expansion or contraction of ocean volume due to temperature and salinity changes. Argo network reaches a quasi-global coverage since 2005 with a nominal spatial resolution of 3° by 3°. We consider here also 4 different solutions from SCRIPPS (Roemmich and Gilson 2009), IPRC (http://apdrc.soest.hawaii.edu/projects/Argo/data/Documentation/gridded-var.pdf), JAMSTEC (Hosoda et al. 2008) and EN4 (Good et al. 2013- all available at https://argo.ucsd.edu/data/argo-data-products/). The first three solutions are also used in Chen et al. (2020). All the Argo data have been interpolated over the SCRIPPS spatial domain for consistency (as in Llovel et al. 2019).

Therefore, correcting the GMSL changes by the steric contributions provides an indirect estimate of the barystatic sea level change. This approach is valuable to assess the robustness of GRACE and GRACE-FO data. This method has been used in several previous studies of GMSL change since 2005 at both global scale (Llovel et al. 2014; WCRP Sea Level Budget Group 2018) and regional scales (Llovel et al. 2011; Marcos et al. 2011), and also for periods over the past decades (Llovel et al. 2013). In the mean time, GRACE/GRACE-FO derived barystatic sea level change provides important validations of altimeter and Argo estimates, especially the Argo estimates (Chen et al. 2020; Barnoud et al. 2021).

**Fig. 11:**
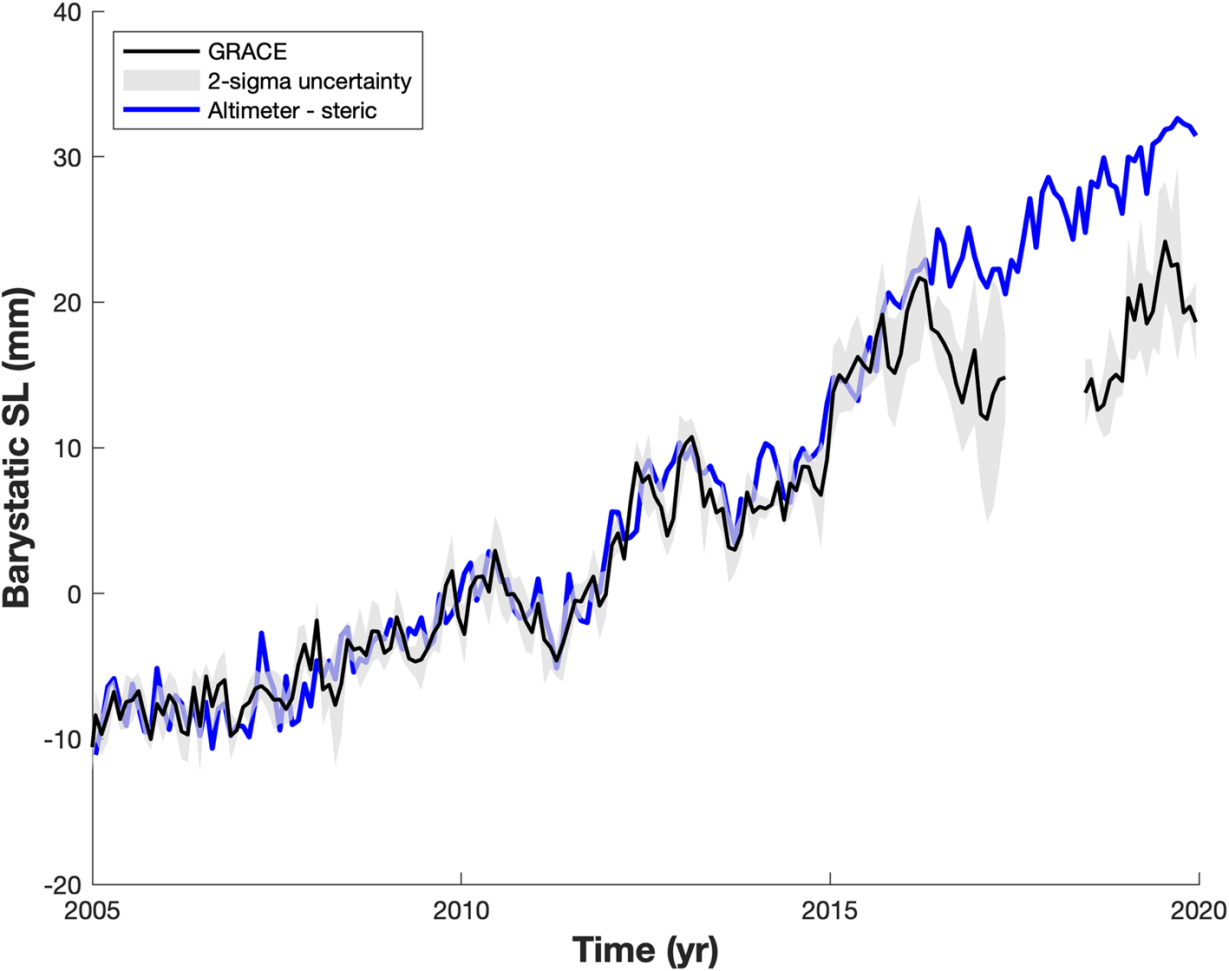
Barystatic sea level change inferred from the mean of the different GRACE and GRACE-FO solutions (black curve with its 2-sigma envelope uncertainty) and from the sea level budget approach (blue curve) over January 2005 and December 2019. Annual signals have been removed

Figure 11 shows the barystatic sea level change (black curve with its uncertainty shown in the gray envelope) from the ensemble mean of the different GRACE/GRACE-FO solutions discussed in 5.1. For consistency, GRACE and GRACE-FO data have been interpolated over the same spatial domain as for Argo gridded products. The blue curve represents the indirect barystatic estimate from altimetry data corrected for Argo-based steric sea level. Comparisons are very good until the end of 2015 meaning that the sea level budget is closed. However, this is no longer the case since around 2016. This disagreement appears coincidentally when GRACE data start to degrade and when parts of Argo floats present positive salinity drifts (Wong et al. 2020; Ponte et al. 2021). A recent study (Barnoud et al. 2021) indicates that instrumental biases in the Argo salinity data can explain about 40% of the discrepancies (since 2016), and the wet tropospheric correction uncertainty of the Jason-3 radiometer (Jason-3 was launched in early 2016) only plays a minor role. Leakage biases in the GRACE/GRACE-FO mascons-based ocean mass estimates can be another major error source. Further investigations are needed to fully understand the causes of the discrepancies.

## Solid Earth Mass Change from GRACE/GRACE-FO

### The Earthquake Cycle

Through its worldwide coverage of the Earth’s major plate boundaries and its sensitivity to mass redistributions at all depths, GRACE has proved a unique tool for studying great earthquakes, especially at subduction boundaries, which record the largest and most devastating events. Earthquakes redistribute mass through the displacement of the density interfaces (mainly the crust surface and base) and the rocks density changes induced by the sudden plates motion. Co-seismic gravity variations associated with 7 recent large events have been detected: the Mw 9.1 Sumatra 2004, the Mw 8.6 Nias 2005, the Mw 8.5 Bengkulu 2007, the Mw 8.8 Maule 2010, the Mw 9.1 Tohoku-Oki 2011 earthquakes, the Mw 8.6/8.2 2012 Sumatra/Indian Ocean doublet, and a deep-focus event: the Mw 8.3 2013 Okhotsk earthquake at 610 km depth (Han et al. 2006; Chen et al. 2007a, b; Panet et al. 2007; de Linage et al. 2009; Heki and Matsuo 2010; Cambiotti and Sabadini 2013; Chao and Liau 2019). The GRACE signal has revealed the importance of co-seismic crustal dilation of the upper plate, leading to a decrease of gravity as a predominant signal (Han et al. 2006). Although crucial for the assessment of seismic hazard, the distribution of the co-seismic slip and the extent and geometry of the ruptured fault plane of such events remain difficult to determine unambiguously from seismic data and geodetic ground networks. The latter are indeed often too distant from the epicentral area, a limitation that can be overcome by the homogeneous spatial coverage of satellite gravimetry especially for undersea events.

For the Mw 9.1 Sumatra 2004, an analysis of the GRACE spatial gravity gradients thus clearly delineated the fault line (Wang et al. 2012a). The current resolution of GRACE is still insufficient to decipher the detailed slip distribution of earthquakes as the Maule 2010 or the Tohoku 2011 one (Wang et al. 2012b, 2012c), but the fault geometric parameters and the average slip can be constrained (Wang et al. 2012b; Dai et al. 2014, 2016). For instance, in the case of the 2011 Tohoku earthquake, the GRACE-based slip orientation appeared tilted as compared to the GPS/seismic determination, and its mean location shifted southwest (Dai et al. 2016). This was shown consistent with a much broader pattern of deformation offshore and at depth than previously known, where the spatial extent of the GRACE co-seismic signal largely exceeded that obtained from slip distributions based on surface displacement and tsunami data (Panet et al. 2018). GRACE has the unique ability to characterize the full magnitude of an earthquake and quantify its entire seismic moment, including all of the slowest components. This way the ultra-long seismic periods of the 2004 Sumatra earthquake have been confirmed, beyond the maximum timescale of a few hundred seconds of most classical seismological inversions (Han et al. 2013).

Understanding the deformation processes at plate boundaries also requires to consider the coupling between the seismic slip and the viscous mantle. This coupling plays an important role in the stress redistributions near the faults, thus in the assessment of the seismic hazard. It is manifested in the spatially distributed viscous mantle relaxation after an earthquake, which relative importance as compared to localized continued aseismic slip after the rupture (afterslip) has been debated for decades (Bürgmann and Dresen 2008; Wang et al. 2012a, b, c, d; Rollins et al. 2015).

Thanks to its homogeneous space–time coverage, GRACE provides unique information on these post-seismic processes, clarifying ambiguities from GNSS observations when they are too distant from the ruptured zone. At the regional scales, the mantle viscous flow after recent large events should indeed appear as a broadscale gravity increase around the ruptured zone (e.g., Einarsson et al. 2010; Panet et al. 2010), whereas the gravity signature of afterslip is expected to be relatively close to the co-seismic one, depending on its depth (Broerse et al. 2015). By detecting different spatial structures of gravity variations at shorter and longer timescales, GRACE has thus shown that both processes have been ongoing after the 2004 Sumatra, 2010 Maule and 2011 Tohoku earthquakes (Tanaka and Heki 2014). For the 2004 Sumatra–Andaman earthquake, the modeling of the observed broadscale gravity increase around the trench in terms of viscous mantle relaxation has provided constraints on transient rheologies in the asthenosphere (Han et al. 2008; Höchner et al. 2011) and suggested low Maxwell viscosities (of order 10^19^ Pa.s) in the underlying upper mantle (Panet et al., 2010), alternatively interpreted in terms of upward diffusion of supercritical water (Ogawa and Heki 2007). These results have been further specified by combining GRACE with surface deformation data, which exhibit a different sensitivity to the mass displacements as a function of depth, ruling out afterslip as the predominant deformation mechanism in the first year following the rupture as well as purely Maxwellian viscosities for the upper mantle, in the case of the 2004 Sumatra earthquake (Panet et al. 2010; Höchner et al. 2011).

Finally, GRACE has led to unexpected observations in recent years. First, post-seismic gravity variations largely exceeding the co-seismic ones have been detected in the case of two earthquakes doublets: the 2006/2007 Mw 8.3/8.1 Kurils earthquakes and the 2009 Mw 8.1 Tonga earthquakes. Although their co-seismic signal was too small to be detected by GRACE, a significant long-term post-seismic gravity variation has been observed in both cases (Han et al. 2016, 2019). Second, a coherent pattern of gravity variations in the months before the 2011 Tohoku-Oki earthquake has been observed and interpreted as related to deeper motions preceding the rupture (Panet et al. 2018). Recently, these results have been corroborated in an independent analysis of GNSS observations (Bedford et al. 2020). Thirdly, GRACE gravitational measurements have been used to detect tsunamis after some major earthquakes during the GRACE period (Ghobadi-Far et al. 2020). Thus, GRACE satellite gravimetry opens new windows for looking into seismic cycle processes.

### GIA and the Earth's Rheology

The Earth deforms over a wide range of timescales due to the rheological properties of its constituent materials, and GRACE contributes to a better knowledge of the mantle viscosity, a key parameter. In addition to controlling the stress distribution in the lithosphere at plate boundaries or in their interior, the mantle viscosity also controls the patterns of the convective flows deep inside the Earth, as well as the Earth’s deformations in response to various forcings. These forcings include the stress variations due to the water load applied at the surface or due to the earthquakes’ sudden motions. The viscosity of the mantle remains difficult to determine only from laboratory experiments on the deformations of mantle minerals, which call for appropriate conditions of pressure and temperature. At the geological timescales, constraints can be obtained from the comparison of surface geophysical observations (as plate motions, static gravity, heat flow) with mantle flow models (King 1995). At shorter timescales, when combined with complementary geophysical observations, GRACE observations of the relaxation signals in the gravity field after excitations such as earthquakes or water/ice-mass redistributions, can provide important information on the Earth's rheological properties.

The major relaxation signal in response to a water/ice load in the GRACE time-varying gravity field corresponds to the long-term gravity trends reflecting the still on-going viscous mantle relaxation in response to stress variations from the melting of the Pleistocene glaciers. It has been detected from GRACE in Northern America, in Fennoscandia and in Antarctica (Tamisiea et al. 2007; Paulson et al. 2007b; Riva et al. 2009; Steffen et al. 2010; van der Wal et al. 2011). By modeling this Glacial Isostatic Adjustment (GIA) signal, inferences have been obtained on the lithospheric thickness, on the upper and the lower mantle viscosity in Northern America, Fennoscandia and Canada (Paulson et al. 2007a, 2007b; Tamisiea et al. 2007; Steffen et al. 2010; van der Wal et al. 2011; Sasgen et al. 2012). Furthermore, the pattern of the GRACE geoid rates over Northern America has provided new insights on the geometry of the former Laurentide ice sheets, shown to comprise two domes (Tamisiea et al. 2007). A major challenge of GIA modeling is indeed to jointly improve our knowledge of the Earth’s rheology together with that of the ice model, as GRACE is sensitive to both (Steffen et al. 2012), and test or refine the existing ice models for the glaciation history. This is important in order to reduce the current uncertainties in the GIA models, which are used in order to separate the GIA signal from those of the present-day climate evolution in sea level observations or over the ice sheets. More recently, the longer time series of available GRACE observations have made possible to detect GIA signals of smaller magnitude and smaller spatial extent, as that of the Svalbard-Barents-Kara Ice Sheet (Root et al. 2015). Modeling of the GRACE signal constrained the upper mantle viscosity in the considered area (Rovira-Navarro et al. 2020), and enabled to discriminate between different models of ice loading history (Root et al. 2015). By showing that regional ice loading models lead to a better fit of the gravity rates than global ice loading models such as ICE-5G (Peltier 2004), GRACE is contributing to a better understanding of the deglaciation history of the Barents Sea.

At shorter timescales, the study of smaller GIA signals as the response of the low-viscosity asthenosphere to ice thickness changes in the last centuries have remained beyond the reach of GRACE. However, constraints on the transient visco-elastic rheology of the asthenosphere at subannual timescales have been obtained by focusing on the mantle response to seasonal water loads, in a combination of GRACE and GPS data (Chanard et al. 2018). Using GRACE to constrain the hydrological water load, the comparison of the modeled and observed GPS horizontal motions allowed to put a lower bound of 5 × 10^17^ Pa.s to the asthenospheric viscosity for global seasonal signals.

## Potential Detection of Deep Earth Signals by GRACE/GRACE-FO

The 2D assumption in GRACE/GRACE-FO mass inversion attributes the observed gravity change to contributions from mass variations on the Earth surface. This is quite reasonable considering that at the studied temporal and spatial scales, mass variations in the climate system (atmosphere, ocean, hydrosphere, and cryosphere) and deformations of the solid Earth are the exclusive or dominant contributors to the observed gravity change. However, interactions between the core and mantle may also affect the time-variable gravity field at interannual, decadal, and long-term time scales.

Core-mantle coupling has long been regarded as the major driving force of the strong decadal variation in length-of-day (LOD) (Hide et al. 1993). LOD also exhibits a persistent quasi six-year oscillation (SYO, at period of ~ 5.9 years) that is linked to interactions between the core and mantle (Mound and Buffett 2006; Holme and de Viron 2013). Similar SYOs are discovered in polar motion (Chen et al. 2019) and global GNSS surface deformation observations (Watkins et al. 2018; Ding and Chao 2018) as well, which are both believed to be from the same origin in deep Earth. Ding and Chao (2018) found clear evidences of the SYO in both radial and horizontal components of global GNSS deformation data, which appear consistent with a westward propagating wave of deformation in degree-2, order-2 ($Y_{2,2}$) sectoral spherical harmonic pattern. A conceptual explanation of the observed SYO signals in $Y_{2,2}$ and LOD is the gravitational coupling between the mantle and inner core, associated with a quasi six-year axial torsional libration of the inner core controlled by the sectoral $Y_{2,2}$ density anomalies, or the equatorial ellipticities, in the inner core and the lower mantle (Ding and Chao 2018).

**Fig. 12:**
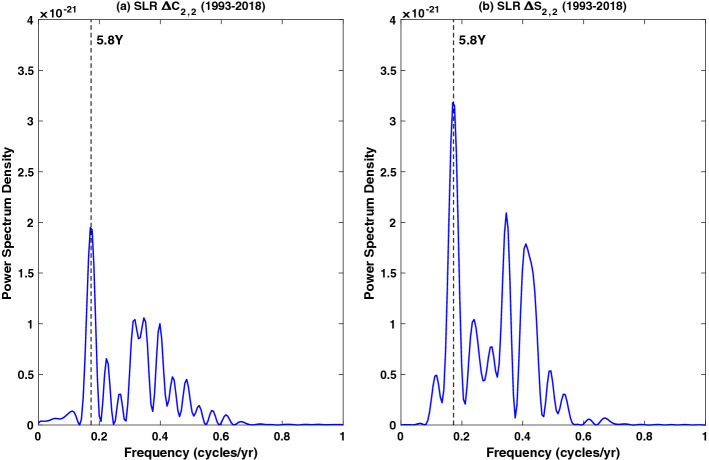
Power spectrum densities of SLR observed **a** $\Delta C_{2,2}$ and **b** $\Delta S_{2,2}$ SH coefficients for the period January 1993–December 2018, provided by the Space Research Institute, Austrian Academy of Sciences. The 5.8-year peaks are marked by the vertical dashed lines

If that is the case, one would easily speculate that similar SYO likely exists in the degree-2 order-2 SH coefficients ($\Delta C_{2,2}$ and $\Delta S_{2,2}$) of the time-variable gravity field as well. This speculation was confirmed by a recent study (Chao and Yu 2020) by analyzing SLR $\Delta C_{2,2}$ and $\Delta S_{2,2}$ variations over the period 1992–2018 and the shorter series from GRACE for 2002–2017. Figure 12 shows the power spectrum densities (PSD) of SLR observed $\Delta C_{2,2}$ and $\Delta S_{2,2}$ SH coefficients for the period January 1993 to December 2018, provided by the Space Research Institute, Austrian Academy of Sciences (available at http://geodesy.iwf.oeaw.ac.at/d_slr_monthly.html) (Maier et al. 2012). To better isolate interannual oscillations, only signals with periods between 2 and 10 years have been retained in the monthly $\Delta C_{2,2}$ and $\Delta S_{2,2}$ series using band-pass filtering, before computing the PSD. Consistent with Chao and Yu (2020), both $\Delta C_{2,2}$ and $\Delta S_{2,2}$ show a clear peak at ~ 5.8 years (marked by the vertical dashed lines). Although there are still many uncertainties and the results need to be further quantified and validated, the interesting findings shed important light on the possible dynamic mechanisms involved in the process, and are anticipated to instigate further studies.

The extended record of GRACE/GRACE-FO time-variable gravity solutions offer another independent means to study the SYO in $\Delta C_{2,2}$ and $\Delta S_{2,2}$ variations. There could be other interannual oscillations in the time-variable gravity field, which might be related to dynamic processes in the core and mantle. A good understanding of these processes and corresponding gravitational changes depends on both continuous accumulation of satellite gravimetry observations with improved accuracy and good independent knowledge of surface mass variations from other techniques or model predictions. Integration of satellite gravimetry and other geodetic observations (e.g., GNSS deformation) can help to improve the understanding of connections between time-variable gravity field and core-mantle interactions.

## Discussion

GRACE/GRACE-FO satellite gravimetry has opened up a new avenue of opportunities for studying large-scale mass redistribution and transport in the Earth system. Over the past 18 years, GRACE/GRACE-FO time-variable gravity measurements have been widely used to study different components of our home planet system, and quantify different variables of the global water cycle (when combined with other available data and/or model predictions). GRACE/GRACE-FO’s tremendous success has been demonstrated by well over two thousand peer-reviewed journal articles based on GRACE/GRACE-FO measurements (a list of GRACE/GRACE-FO related articles is compiled at http://www-app2.gfz-potsdam.de/pb1/op/grace//references/sort_author.html). Since the launch of GRACE in 2002, continuous improvements in the background geophysical models, understanding and analysis of the GRACE/GRACE-FO instrument data, and gravity field determination procedure have significantly improved the accuracy of GRACE/GRACE-FO time-variable gravity solutions.

Among the major challenges discussed in the paper, the coarse spatial resolution and leakage error of GRACE/GRACE-FO gravity solutions are the key factors affecting most GRACE/GRACE-FO applications in related fields. The spatial resolution is a fundamental limitation of GRACE/GRACE-FO satellite gravimetry related to satellite orbit configurations (e.g., inter-satellite distance and satellite altitude). This is not expected to change for GRACE/GRACE-FO, unless for future generations of satellite gravity missions with different configurations of satellite pairs (e.g., multi pairs with different inclinations). The non-uniqueness of mass inversion from GRACE/GRACE-FO gravity solutions is another major factoring limiting GRACE/GRACE-FO applications. Other independent data or model estimates are needed for separating different sources in GRACE/GRACE-FO observed mass change. Due to the non-uniqueness restraint, it is impossible for GRACE/GRACE-FO to directly measure mass changes in the deep Earth. However, certain interannual oscillations in the observed gravity field are likely connected to interactions between the core and mantle.

Extending satellite gravity observations is essential for better understanding mass redistribution and transport in the Earth system, especially the long-term variability of water mass change in the hydrosphere, ocean and cryosphere. GRACE/GRACE-FO satellite gravimetry plays an important role in helping understand climate change (e.g., ice melting, sea level rise, and groundwater depletion), and offers a means for monitoring the severities and scopes of extreme climate events, such as major droughts and floods from a completely new perspective. With a nominal mission lifetime of five years (same as GRACE), GRACE-FO has been in orbit for over 3 years, and is expected to well exceed the nominal mission lifetime based on satellite and instrument design and the influence of solar activity on the atmospheric-induced decay of the spacecraft (Tapley et al. 2019). The extended record of GRACE/GRACE-FO gravity series, with expected continuous improvements in the coming years, will lead to a broader range of applications and improved our understanding of both climate change and the Earth system.
